# Attention Deficit/Hyperactivity Disorder (ADHD)-Related Strengths in Adults: A Scoping Review

**DOI:** 10.1177/10870547261425737

**Published:** 2026-03-26

**Authors:** Rivqa B. Rafael, Hongwei Jia, Melissa Rouel, Bethany M. Wootton, Deborah Mitchison

**Affiliations:** 1 University of Technology Sydney, Ultimo, NSW, Australia

**Keywords:** adult ADHD, strengths, neurodiversity, review

## Abstract

**Objectives:**

Increasingly, people with attention deficit/hyperactivity disorder (ADHD), their clinicians, and others have called for strengths-based approaches in the understanding of ADHD. A comprehensive and systematic review of ADHD-related strengths has not previously been published. Therefore, this study aimed to conduct a scoping review to determine the extent and type of empirical qualitative and quantitative research about strengths that may be related to ADHD in adults.

**Methods:**

MEDLINE, Embase, PsycINFO, and Scopus were searched for published research. ProQuest Dissertations & Theses Global, PsyArXiv, and other websites were searched for unpublished studies or grey literature.

**Results:**

A total of 125 studies were included (61 qualitative studies, 59 quantitative studies, 5 mixed methods studies). Most studies examined ADHD-related strengths a-priori (*n* = 88; 70%). The majority studied ADHD strengths or experiences in general terms (*n* = 68; 54%), rather than focussing on a specific strength or characteristic. It was observed that typical ADHD characteristics were sometimes perceived or redefined as strengths: *interest-based attention* or positive differences in attentional ability (*n* = 52; 42%), *energy* or positive differences in activity levels (*n* = 33; 26%), and *adaptive risk-taking* or positive aspects of impulsivity (*n* = 19; 15%). Other strengths identified across studies included: creativity (*n* = 82; 66%), prosocial attributes (*n* = 39; 31%), entrepreneurship (*n* = 14; 11%), resilience (*n* = 16; 13%), flexibility (*n* = 14; 11%), uniqueness (*n* = 16; 13%), and other characteristics (*n* = 24; 19%).

**Conclusions:**

Further research will help mental health professionals support people with ADHD through their challenges while helping them develop and utilise their strengths in contexts where they are most likely to flourish.

**Pre-registration:**

https://osf.io/43nd9.

## Introduction

The challenges experienced by people with attention deficit/hyperactivity disorder (ADHD) are well documented, including stigma (both external and internalised) and poorer outcomes than peers without ADHD across quality-of-life measures (Faraone et al., 2021). Biomedically, ADHD is conceptualised as a neurodevelopmental condition, comprising challenges with hyperactive and impulsive behaviours and/or maintaining attention relative to norms (American Psychiatric Association, 2022). Yet alternatives to this perspective are emerging in the literature, which consider ADHD as a difference associated with strengths as well as challenges, rather than a disorder (Bölte et al., 2021; Sonuga-Barke et al., 2023). With recent clinical guidelines calling for strengths-based methods in ADHD assessment and support (ADHD Guideline Development Group [AGDG], 2022), it is timely to further consider strengths in relation to the phenomenology of ADHD.

Historically, ADHD has been conceptualised and studied through a deficit-based lens (Champ et al., 2021; Chaulagain et al., 2023). For example, in a large meta-review of systematic reviews on ADHD, most of the 231 included papers focussed on interventions; other common topics included comorbidities, prognosis, and risk factors (Chaulagain et al., 2023). Only three reviews included by Chaulagain et al. (2023) considered positive aspects of ADHD among adults, adolescents, and children and their parents, respectively (Bjerrum et al., 2017; Eccleston et al., 2019; Wong et al., 2018). Based on their systematic review of 10 qualitative studies on adults’ lived experience of ADHD, Bjerrum et al. (2017) synthesised a finding of “Adults with ADHD are creative and inventive,” with a moderate ConQual score (credibility downgraded one level due to equivocal findings). Similarly, Eccleston et al. (2019) systematically reviewed qualitative studies of adolescents’ ADHD experiences, noting that some participants considered their ADHD characteristics, such as being energetic, creative, and fun, to be personal strengths. In a review of 101 articles on perceptions of ADHD, Wong et al. (2018) highlighted studies wherein children and adolescents with ADHD and their parents identified positive attributes related to ADHD, such as increased energy and extroversion. Although each of these reviews mentioned potential ADHD strengths, none focussed solely on positives; indeed, mentions of strengths were a small section of included studies.

Notably, a scoping review that aimed to identify potential strengths of ADHD alongside its challenges could not meet its objective, finding no studies on strengths and uncovering only studies relating to disability and impairment (de Schipper et al., 2015). This outcome may have been due to methodological factors, as only a random sample of search results were evaluated in this study. However, it is a poignant illustration of the infancy of strengths-based approaches in ADHD. Ten years on, and with increased calls for strengths-based methods, the context and findings may be different.

Although not specific to ADHD, strengths-based methods in psychological therapies engage with a person’s abilities to gain a fuller perspective of their life and improve their quality of life by further developing these positive attributes as well as leveraging them to help manage their challenges (Flückiger et al., 2023). A meta-analysis examining strengths-based methods in psychotherapy sessions across several modalities found that such techniques are associated with a small but significant improvement in therapy outcomes beyond standard treatment (Flückiger et al., 2023). While frameworks exist for conceptualising strengths, such as character strengths and virtues (CSV; Peterson & Seligman, 2004), Flückiger et al. (2023) advocate for a client-centred approach to identifying and working with individuals’ strengths. Clinically, this involves collaborating with clients to understand their values and perspectives about strengths, including cultural considerations. In a research context, this approach is analogous to a participatory research framework, where people with lived experience are included in the design and implementation of research (Vaughn & Jacquez, 2020). As ADHD is present across cultures, genders, cognitive abilities, and the lifespan, and has no agreed-upon aetiology or theoretical framework (Champ et al., 2023; Faraone et al., 2021), it must be concluded that despite their challenges, people with ADHD can vary in their strengths as much as people without ADHD. Thus, when implementing strengths-based methods, an individual’s positive attributes must all be considered, whether connected to ADHD or not.

Nonetheless, studying strengths that may be inherent to ADHD can add to previous literature, determine whether any strengths—or their mechanisms—are unique to ADHD, and contribute to the evidence base for strengths-based frameworks. Many previous studies, including the aforementioned reviews (Bjerrum et al., 2017; de Schipper et al., 2015; Eccleston et al., 2019; Wong et al., 2018), have focussed on strengths or positives related to ADHD, whether or not they are unique to ADHD. Bjerrum et al. (2017) highlighted findings that creativity in the context of ADHD involved making quick connections and seeing things others missed, which may suggest some differences from non-ADHD creativity. One qualitative study of ADHD strengths found that Peterson and Seligman’s (2004) CSV framework did not include all the themes they identified (Sedgwick et al., 2019). Sedgwick et al. (2019) suggested that the themes that overlapped with the CSV may be more general strengths (e.g., curiosity), whereas those that did not represented ADHD-specific strengths (e.g., hyperfocus).

Researching the positives of ADHD is also a means for engaging with pre-existing strength-based frameworks for understanding ADHD. Prominent among these is the neurodiversity framework or paradigm, which argues that, like biodiversity in nature, variation in cognitive and neurological patterns is positive for individuals and the human species as a whole (Dwyer, 2022; Singer, 1998). Some advocates argue that the challenges accompanying autism, ADHD, and other neurobiological differences are entirely due to societal factors, whereas others acknowledge the role of both inherent and societal factors (Dwyer, 2022). However, proponents agree that “curing” such differences should never be the goal; rather, this diversity should be accepted, even celebrated (Dwyer, 2022).

In a neurodiversity paradigm (Dwyer, 2022; Singer, 1998), inattention (and its paradoxical counterpart, hyperfocus) can be reframed as *interest-based attention*, while *intensity* can overlap with hyperactivity and impulsivity as well as hyperfocus experiences (Bertilsdotter Rosqvist et al., 2023a). Such terminology does not align with diagnostic criteria, instead offering a holistic framework for understanding ADHD experiences. Self-determination theory has also been posited as a framework for interest-based attention (Champ et al., 2023). In this theoretical application, Champ et al. (2023) suggest that the neurobiological differences seen in ADHD result in strong intrinsic motivation for activities of interest, up to and including hyperfocus experiences. Conversely, motivation is harder to access when the activity is not interesting to the individual with ADHD, even if deemed important. Although people without ADHD also experience such variations in motivation, they may be more pronounced among those with ADHD. Although this theoretical application is yet to be tested empirically, it is notable as it posits a neurobiological mechanism for ADHD strengths, in addition to its challenges.

Recently, there have also been calls for a neurodivergent pride movement, analogous to queer pride, to foster positive autistic, ADHD, and other identities (Chapman & Botha, 2023). Such advocacy functions to reduce internalised stigma for those with ADHD and improve self-esteem. Empirical evidence for ADHD-related strengths would be useful for this purpose. However, clinicians and lived experience advocates share concerns that neurodiversity paradigms, if taken to extremes, may also perpetuate challenges (Bertilsdotter Rosqvist et al., 2023b; Dwyer, 2022). Although recent popular culture occasionally frames ADHD as a “superpower,” this may elide challenges (albeit often created by neurotypical expectations of mainstream society), while increasing pressure placed on people with ADHD (Bertilsdotter Rosqvist et al., 2023b). The “superpower” discourse may also increase barriers to support when needed (Dwyer, 2022). To avoid these issues, any framework for appreciation of the positives of ADHD must also integrate an understanding of related challenges.

One proposed method to reconcile neurodiversity paradigms with the biomedical model is the World Health Organisation International Classification of Functioning Disability and Health (ICF; Bölte et al., 2021). The ICF is a comprehensive framework for understanding ability and disability in the context of an individual’s environment, although they are acknowledged to be lengthy and impractical for clinical use (Bölte et al., 2021). ICF “core sets,” or lists of relevant categories for specific conditions or differences, make the framework more usable (Bölte et al., 2018, 2021). An ICF core set for ADHD was developed through a series of multinational studies (Bölte et al., 2018; de Schipper et al., 2015; de Schipper et al., 2015; Mahdi et al., 2017, 2018). With 72 categories overall (47–55 for specific age groups) such as “energy and drive functions” and “solving problems,” the ADHD core set requires further development to be useful to clinicians (Bölte et al., 2018, 2021).

The ICF offers one solution to a concern apparent in the research and clinical practice guidelines: unless we, as clinicians and researchers, actively seek out strengths and positives, they may not become apparent to us. Selecting appropriate objectives, followed by complementary methodologies, are important first steps in this regard (Lyon et al., 2017). Previous research into potential ADHD strengths may provide a useful starting point. Although not captured by earlier scoping reviews (Champ et al., 2021; de Schipper et al., 2015), some research has investigated ADHD-related strengths. Two topics are relatively more researched—creativity and hyperfocus.

The most extensively studied potential ADHD strength is creativity, usually operationalised as convergent or divergent thinking or creative achievement (Hoogman et al., 2020; Paek et al., 2016). A meta-analysis of 89 studies (30 specific to ADHD) on associations between ADHD, depression, and anxiety and “little c” or everyday creativity, found no significant relationship (Paek et al., 2016). However, the authors noted that this may be due to the heterogeneous nature of the included studies, and moderating factors, such as degree of impairment. Testing for publication bias also indicated that studies with negative findings may not have been published. Conversely, a systematic review reported an association between divergent thinking and subclinical ADHD in many of the 31 included studies, but no associations with convergent thinking or clinical ADHD (Hoogman et al., 2020). They also summarised neuroimaging research, which indirectly showed a relationship between ADHD and creativity, but noted that direct evidence is lacking. Although Hoogman et al. (2020) did not include a meta-analysis, they noted heterogeneity and poor quality of the included studies, as did Paek et al. (2016). More rigorous research is needed to resolve these inconsistencies and determine whether the brain differences seen in ADHD are related to creativity.

One narrative review has examined hyperfocus, a phenomenon associated with autism and schizophrenia, as well as ADHD (Ashinoff & Abu-Akel, 2021). The authors collated different definitions of hyperfocus, ranging from the more positive concept of *flow* to “being in the zone,” which was seen as less uniformly positive by participants across the included studies. Definitions also varied based on the condition or neurobiological difference being studied. Although this review briefly mentioned the potential positives of hyperfocus in the context of ADHD, its predominant concerns were the neurobiology and classification of hyperfocus, not its benefits.

While these reviews covered some specific ADHD-related strengths (Ashinoff & Abu-Akel, 2021; Hoogman et al., 2020; Paek et al., 2016), research about ADHD-related strengths has never been systematically collated, and few reviews have focussed exclusively on adults. Adults with ADHD were selected as the target population for the present study both due to feasibility concerns, and to ensure maximal inclusion of personal lived experiences. As seen in a review of articles on the perceptions of children and adolescents with ADHD and their parents, relatively few studies elicit children’s perspectives (Wong et al., 2018). Moreover, it was anticipated that adults would be better positioned to reflect on their lives and experiences and distinguish between potential innate ADHD strengths and strengths gained from managing their ADHD-related challenges. Lastly, it was planned to limit the population to adults due to feasibility concerns, as the volume of articles to be screened was expected to be large due to the general nature of the required search terminology.

When considering the importance of lived experience, it is important to note that there may be strengths that are related to or even intrinsic to ADHD, yet may be difficult to study empirically, even as they are known to people with ADHD and those close to them. Psychological research has been critiqued for its gender- and race-based biases in relation to which forms of knowledge are privileged (Eagly & Riger, 2014; Teo, 2022), and who experiences epistemic injustice, or discrimination based on a person or minoritised group’s credibility as a holder of knowledge (Byskov, 2021). The lived experience of the first author (for a positionality statement, see Methods) suggested that bias across many axes, as well as pragmatic considerations, may limit the study of some strengths, particularly outside qualitative research. To mitigate epistemic injustice, it is critical to collate all forms of knowledge on this topic, from empirical research to laypeople’s discussions, and to consider the full range when generating hypotheses and uncovering gaps in the literature. As researchers plan and conduct strengths-based research to broaden our ways of knowing, as well as what we know, clinicians and people with ADHD should be informed by the available evidence, with an emphasis on the potentiality of strengths. Although constructs such as correlation, predictors, and outcomes are of great importance for improving the lives of people with ADHD, it is equally important to begin by identifying as many potential strengths as possible. As a starting point in this endeavour, this study aimed to address the limitations in existing literature by conducting a scoping review to determine the extent of empirical qualitative and quantitative research about strengths that may be related to ADHD in adults. With no a-priori hypotheses, the current study aimed to answer the question: Which personal strengths have been empirically studied in adults with ADHD to date?

## Methods

This review was pre-registered on OSF (https://osf.io/43nd9), and conducted in accordance with the JBI methodology for scoping reviews (Aromataris et al., 2024) and the Preferred Reporting Items for Systematic Reviews and Meta-Analysis extension for scoping reviews (PRISMA-ScR; Tricco et al., 2018). A scoping review was selected as the most appropriate form of evidence synthesis for this topic due to the anticipated heterogeneity of research and breadth of the topic (Munn et al., 2022). More specifically, given the lack of extant reviews on the topic, mapping the breadth of the field was considered a more appropriate first step, to precede other forms of evidence synthesis, such as a systematic review or meta-synthesis. For this reason, considering outcomes (i.e., whether any strengths identified were indeed caused by or correlated with ADHD) and evidence quality was thought to be pre-emptive. As scoping reviews typically do not chart or extract such data (Pollock et al., 2023), this methodology was determined to be the most appropriate. Further, a scoping review was considered to best allow for different sources (e.g., peer-reviewed articles, theses, and preprints) and types of evidence (e.g., qualitative and quantitative studies with varying methodologies) on a range of potential strengths. The “iterative and flexible” (Peters et al., 2021, p. 3) nature of scoping reviews was also noted as a potential benefit, given the paucity of research and lack of clear operational definitions.

### Search Strategy

The search strategy aimed to locate published and unpublished studies. An initial limited search of PubMed and PsycINFO was undertaken to identify relevant articles. Titles and abstracts of relevant articles, and their index terms, were used to develop a full search strategy (Supplemental File 1). The search strategy, including all identified keywords and index terms, were adapted for each included database and/or information source. The reference lists of key reviews and clinical guidelines were hand searched for additional studies (Supplemental File 1).

MEDLINE (Ovid), Embase (Ovid), PsycINFO (EBSCO), and Scopus were searched for published research. ProQuest Dissertations & Theses Global, PsyArXiv, and lived experience or advocacy organisation websites were searched for unpublished studies or grey literature (Supplemental File 1). Relevant websites were identified with an exploratory Google search (first five pages of results). The ProQuest search strategy is delineated in Supplemental File 1. PsyArXiv and other websites were searched manually for relevant articles. The initial search was conducted from 8 April 2024 to 9 September 2024; an updated search was conducted on 4 February 2025.

### Eligibility Criteria

#### Participants

Studies were included if some or all participants were aged 18 years or older, and had an ADHD diagnosis (whether defined by self-report, clinician interview, or other methods), or ADHD characteristics as identified by the study, regardless of whether co-occurring conditions were present (Supplemental File 2). This wider definition of ADHD was selected in favour of using stricter diagnostic criteria to maximise the breadth of included studies. It was anticipated that many studies would not be able to confirm diagnoses (e.g., anonymous online surveys). Studies were also included if participants did not have ADHD, but referred to adult subjects with ADHD (e.g., where clinicians or other third parties described strengths of adults with ADHD). Studies where all participants or subjects were below the age of 18 years were excluded.

#### Concept

Studies were included if they involved qualitative or quantitative research into potential strengths that may be related to ADHD, whether general or specific to a single strength and regardless of how much detail was provided (Supplemental File 2). Studies were included if researchers and/or participants mentioned one or more potential ADHD-related strengths, positive attributes or aspects, or achievements deriving from ADHD. Studies were included if they covered both strengths and challenges, but excluded if they did not mention strengths that may relate to ADHD. For example, studies of mediating or moderating factors were excluded. Further, studies were included if they referred to potentially positive aspects of ADHD traits or phenomena (e.g., impulsivity; hyperfocus) even if they also referred to negative aspects of these traits, but excluded if focussed only on the negative aspects of these traits. Studies were included based on their topic or hypothesis, rather than outcomes (e.g., studies of creativity in ADHD were included whether an association was observed or not). Conclusions drawn from unexpected results were also included (i.e., where researchers had hypothesised a deficit but encountered a strength, and offered conjecture as to why this might have occurred). Studies were excluded if they mentioned strengths without specifying any connexion with ADHD.

#### Context

Other contextual factors (e.g., gender, geographical location) did not form inclusion or exclusion criteria (Supplemental File 2). Studies published in any language were included if they could be translated into English, or if they included an English-language summary. No date restrictions were used in the search, to provide a full historical account of the topic.

#### Types of Sources

This scoping review considered experimental and quasi-experimental study designs, including randomised and non-randomised controlled trials, within-group pre-post studies, and interrupted time-series studies (Supplemental File 2). In addition, analytical observational studies including prospective and retrospective cohort studies, case–control studies and analytical cross-sectional studies were considered for inclusion. This review also considered descriptive observational study designs including case series, individual case reports and descriptive cross-sectional studies for inclusion. Also considered were qualitative studies, including, but not limited to, designs such as phenomenology, grounded theory, ethnography, qualitative description, action research, and feminist research. Reviews, opinion articles, and other non-empirical works were excluded.

### Study Selection

**Figure 1.**
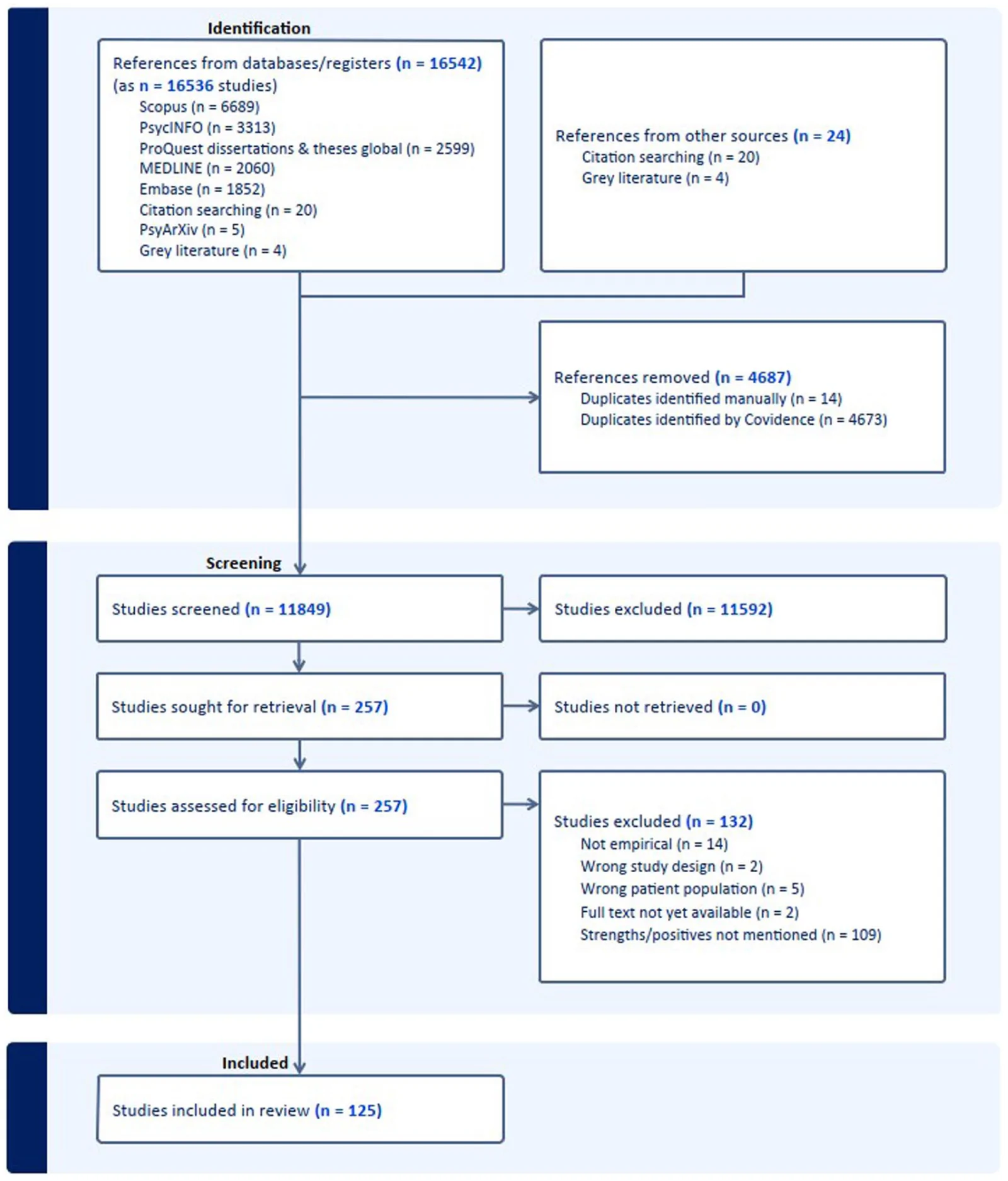
Flow diagram of study inclusion and exclusion.

After the search, all identified citations were collated and uploaded into Covidence (Covidence, 2024) and duplicates (*n* = 4,687) removed. Following a pilot test, titles and abstracts were screened by one reviewer for assessment against the inclusion criteria for the review. A second reviewer screened 10% of the title and abstract initial search results for quality assurance (37/1,125 conflicts were resolved through discussion; 97% agreement). The full text of potentially relevant citations (*n* = 257) was assessed in detail against the inclusion criteria by one reviewer, and a second reviewer screened 10% of the full-text initial search results for quality assurance (8/26 conflicts were resolved through discussion; 70% agreement). Reasons for exclusion of sources of evidence at the full-text screening stage were recorded and reported. Any disagreements that arose between the reviewers at each stage of the selection process were resolved through discussion. Figure 1 presents the results of the search and the study inclusion process in a PRISMA-ScR flow diagram (Tricco et al., 2018).

### Data Extraction

Data were extracted into LibreOffice Calc (LibreOffice, 2024) from included studies by the main reviewer using a data extraction tool developed by the reviewer according to scoping review extraction guidelines (Supplemental File 2; Arksey & O’Malley, 2005; Tricco et al., 2018). The extracted data included specific details about the participants, concept, context, study methods and key findings relevant to the review question. Specifically, strengths mentioned in the results or discussion sections of included studies were extracted, but statistical findings (e.g., correlations, *p* values, or effect sizes) were not. In line with scoping review recommendations, this decision aimed to prevent conclusions from being drawn about correlation or causality (Pollock et al., 2023), to retain a focus on the breadth of the field and hypothesis generation. As scoping review data extraction can be an iterative process (Pollock et al., 2023), some items for extraction were added during the extraction process; these have been noted in Supplemental File 2. If appropriate, authors of studies were contacted to request missing or additional data, where required. In line with the aims of this review and recommended scoping review methodology (Peters et al., 2021), critical appraisal or risk of bias assessment of sources was not performed.

### Data Analysis and Presentation

As a scoping review, this study did not synthesise or aggregate findings (Arksey & O’Malley, 2005), but instead described the state of the research narratively and with use of descriptive statistics. Basic qualitative analysis (Pollock et al., 2023) was conducted using Taguette (Rampin & Rampin, 2021) to categorise the strengths mentioned. This analysis was not intended as definitive thematic analysis; rather, it aimed to create manageable categories for the purpose of this review only. A mixed inductive–deductive approach was used, wherein extracted terminology was initially coded without use of a formal framework. Codes that appeared to fit into existing categories in the literature were then further combined to create the final categories. As some studies used multiple synonyms for some strengths, each category was only counted once per study. Research data were shared on OSF (https://osf.io/43nd9).

### Positionality Statement

The main researcher of this review (RR) has lived experience of ADHD, and clinical and research experience and interest in strengths-based psychology. She acknowledges that her lived experience does not represent all ADHD experiences, but is one of many. She acknowledges that academia, and psychology and related disciplines, have both harmed and helped people with ADHD, among other minoritised people. She seeks to use her privilege afforded by her position, which is directly related to white privilege and class privilege, to redress harm and increase benefit.

## Results

### Study Characteristics

**Figure 2.**
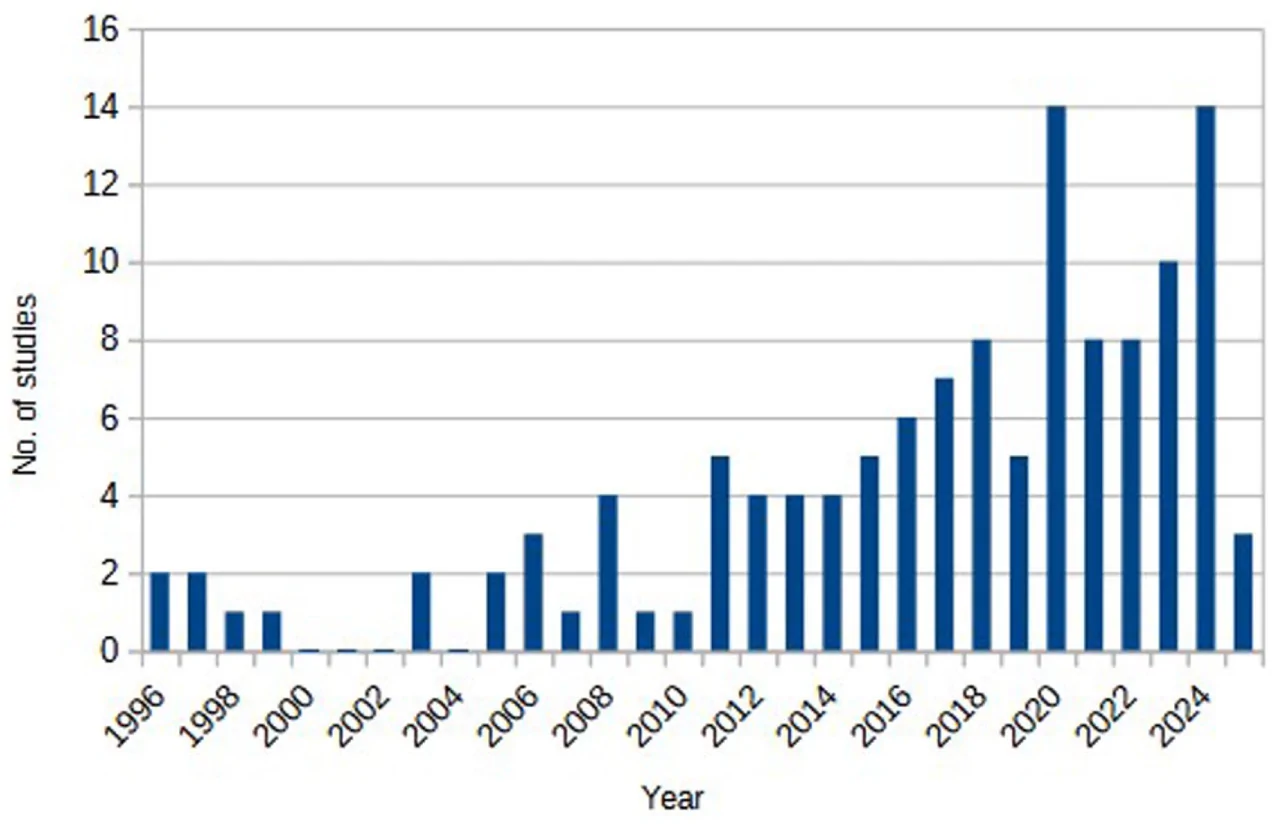
Included studies by year of publication.

In total, 125 studies were included in the review (Table 1). Included studies were published between 1996 and 2025; five studies were preprints (Figure 2). Eleven studies were multinational (9%); the remainder originated from 20 different countries, predominantly the United States (*n* = 59; 47%), the United Kingdom (*n* = 14; 11%), and other Western countries (*n* = 31; 25%). Not including dissertations and preprints, two studies were found in grey literature (Supplemental File 3). There were 61 qualitative and 59 quantitative studies; five studies employed mixed qualitative and quantitative methods (49%, 47%, and 5%, respectively). Of the quantitative studies, 54 (92%) employed a comparison group and two studies had a within-subjects design (3%); one survey of people with ADHD, one clinician survey, and one scale development study with clinician participants had no comparison group.

**Table 1.** Details of Included Studies.

Citation	Country	Study topic	A-priori study of strengths	Study design	Identification of ADHD	Population	*N*	Gender (% women)
Abramowicz (1998)	USA	General	Y	Qual; interviews	Self-reported diagnosis; screened (ADHD Behaviour Checklist for Adults)	Adults with ADHD (successful)	2	50%
ADDitude Editors (2021)	Multinational	General	Y	Qual; survey	Self-identified	ADDitude readers	2,295	NS
ADDitude Editors (2020)	Multinational	General	Y	Qual; survey	Self-identified	ADDitude readers	1977	NS
Alt (1999)	USA	Creativity	Y	Quant; quasi-exp	Self-reported diagnosis; screened (bespoke)	Adults	110	49%
Andreou and Trott (2013)	Greece	Verbal fluency	N	Quant; quasi-exp	Self-reported diagnosis; screened (bespoke)	University students	60	30% (ADHD group)
Anhalt (2015)	USA	General	Y	Qual; interviews	Self-reported diagnosis and/or screened (ASRS-18)	Adults with ADHD	8	38%
Atkins (1997)	USA	General	Y	Qual; interviews	Self-reported diagnosis	Adults with ADHD	7	57%
Barack et al. (2024)	USA	Foraging	Y	Quant; quasi-exp	Screened (ASRS-5)	Representative US sample	457	47%
Barkley et al. (1996)	USA	General	N	Quant; quasi-exp	Verified diagnosis	Young adults	48	36%–39%
Beesley (2024)	USA	Creativity	Y	Quant; survey	Self-identified; screened (ASRS-18)	Adults	334	58%
Belo-Tomic et al. (2021)	Australia	General	Y	Qual; interviews	Other^a^	Young adults with a parent diagnosed with ADHD	4	50%
Bertilsdotter Rosqvist et al. (2023a)	Sweden	General	Y	Qual; autoethnography	Self-identified	Neurodivergent academic researchers	6	NS
Boot et al. (2020)	Netherlands	Creativity	Y	Quant; quasi-exp	Screened (ADHD-RS)^b^	Adults	197	61%–62%
Boot et al. (2017)	Netherlands	Creativity	Y	Quant; quasi-exp	Verified diagnosis	University students	940	67%–72%
Bosworth (2023)	USA	General	Y	Mixed methods; survey, interviews	Self-identified	University students (design) with ADHD	86	NS
Brock (2008)	USA	General	N	Mixed methods; focus groups, interviews	Self-reported diagnosis	Teachers with ADHD	9	89%
Brod, Pohlman et al. (2012)	Multinational	General	N	Qual; interviews and focus groups	Self-reported diagnosis	Adults with ADHD	108	47%
Brod, Schmitt et al. (2012)	USA	General	N	Qual; interviews	Self-reported diagnosis	Older adults with ADHD	24	32%
Brouillard and Byers-Heinlein (2023)	Canada	Vocabulary	N	Quant; quasi-exp	Screened (ASRS-18)	Young adults	391	57%
Button (2019)	USA	General	Y	Qual; interviews	Self-reported diagnosis	Adults with ADHD (workers)	10	50%
Canela et al. (2017)	Switzerland	General	Y	Qual; interviews	Verified diagnosis	Adults with ADHD (outpatients)	32	44%
Cardello and George (2021)	USA	Creativity	Y	Quant; quasi-exp	Screened (ASRS-18)	University students	65	75%
Champ et al. (2024)	UK	General	Y	Qual; interviews	Self-reported diagnosis^c^	Adults with ADHD	11	55%
Chiappa (2003)	USA	General	Y	Qual; interviews	Self-identified; screened (bespoke)	Mental health counsellors diagnosed with ADD	8	50%
Craddock (2024)	UK	General	N	Qual; interviews	Self-reported diagnosis	Women with auADHD	6	100%
Crook and McDowall (2024)	UK	General	Y	Qual; interviews	Self-reported diagnosis	Adults with ADHD	17	53%
Davis (2014)	USA	General	Y	Qual; interviews	Self-reported diagnosis	Women with ADHD and LD (successful)	5	100%
de Schipper et al. (2015)	Multinational	General	Y	Quant; survey^d^	NA	International experts in ADHD	174	67%
Dimic and Orlov (2015)	Finland	Entrepreneurship	Y	Quant; survey	Self-identified	Adults	270	62%
Duke (2023)	Switzerland	Mind wandering	N	Qual; interviews	Self-reported diagnosis	Young adults with ADHD	8	NS
DuPaul et al. (2017)	USA	General	N	Quant; survey	Self-identified	University students (first-year) with ADHD, LD, both, or neither	15,273	44%–57%
Engelthaler (2021)	USA	Social skills	N	Mixed methods; survey, activity, content analysis	Screened (ASRS screener)	University students (psychology)	130	65%–73%
Fleischmann and Fleischmann (2012), Fleischmann and Miller (2013)	Multinational	General	N	Qual; content analysis	Self-reported diagnosis	Adults with ADHD	71	NS
Forbes (2014)	USA	General	Y	Qual; interviews	Self-reported diagnosis	Adults with ADHD (successful)	6	50%
Girard-Joyal and Gauthier (2022)	Canada	Creativity	Y	Quant; quasi-exp	Self-reported diagnosis	Adults	83	76%
Godfrey-Harris and Shaw (2023)	UK	General	Y	Qual; interviews	Self-reported diagnosis	University students (medical) with ADHD	6	67%
Griffin (1996)	USA	Creativity	Y	Quant; quasi-exp	Screened (WURS)	University students (psychology)	135	66%
Grønneberg et al. (2024)	Norway	General	Y	Qual; interviews	Verified diagnosis	Young adults with ADHD	10	50%
Grossman et al. (2015)	Israel	Inattentional blindness	Y	Quant; quasi-exp	Verified diagnosis	University students	32	NS
Grotewiel et al. (2023)	USA	Hyperfocus	Y	Quant; survey	Screened (ASRS-18)	University students	85	62%
Gully (2009)	USA	General	Y	Qual; interviews	Verified diagnosis	University graduates with ADHD (identified as gifted in elementary school)	4	25%
Hammond (2010)	Canada	General	N	Qual; interviews	Self-identified	Adults with ADHD	5	40%
Hansson Halleröd et al. (2015)	Sweden	General	N	Qual; interviews	Verified diagnosis	Adults with ADHD (diagnosed in adulthood)	21	52%
Harris (2020)	USA	General	N	Qual; interviews	Self-reported diagnosis	Adults with ADHD	12	75%
Healy (2006)	USA	General	N	Qual; interviews	Self-reported diagnosis	University graduates with ADHD	7	71%
Hedlund and Jordal (2024)	Sweden	General	Y	Qual; interviews	Self-reported diagnosis	Nurses with ADHD and/or autism	17	94%
Henry and Jones (2011)	USA	General	N	Qual; interviews	Verified diagnosis	Women with ADHD (diagnosed after the age of 60)	9	100%
Hoffman (2020)	USA	General	Y	Qual; interviews	Self-identified	Classical musicians with ADHD	8	50%
Holmes (2005)	USA	General	Y	Qual; interviews	Self-reported diagnosis	Successful adults with ADHD	4	50%
Holthe and Langvik (2017)	Norway	General	N	Qual; interviews	Self-reported diagnosis	Women with ADHD (diagnosed as adults)	5	100%
Holtsclaw (2020)	USA	Empathy	Y	Qual; interviews	Self-reported diagnosis	Middle-aged adults with ADHD	11	82%
Hotez et al. (2022)	USA	General	N	Quant; survey (secondary data analysis)	Self-identified	University students	7,731,640	43%–55%
Hupfeld et al. (2019)	USA	Hyperfocus	Y	Quant; survey	Screened (ASRS screener)	Adults	623	NS
Hupfeld et al. (2024)	USA	Hyperfocus	Y	Quant; survey	Self-reported diagnosis	Adults	347	47%
Fani Isfahani et al. (2022)	Iran	Entrepreneurship	Y	Quant; survey	Screened (ASRS-18)	Iranian entrepreneurs	388	51%
Jones et al. (2023)	USA	Creativity	Y	Quant; quasi-exp	Self-reported diagnosis	Adults	31	48%
Jordan (2020)	USA	Hyperfocus	Y	Quant; quasi-exp	Screened (ASRS screener)	University students	115	73%
Kahn (2007)	USA	Creativity	Y	Quant; quasi-exp	Self-reported diagnosis; screened (bespoke)	Adults	32	31%
Kamath et al. (2020)	USA	Sensory experiences	N	Quant; quasi-exp	Verified diagnosis	Adults	45	49%–51%
Katabi et al. (2023)	Multinational	General	Y	Quant; survey^d^	Self-reported diagnosis	Adults with ADHD	260	48%–75%
Kent (2017)	USA	General	Y	Qual; interviews	Self-reported diagnosis	Adults with ADHD (successful)	8	50%
Karpiuk Vertone (2024)	USA	General	Y	Qual; interviews	Screened (ASRS-18, CAARS, WURS)	Women with ADHD	13	100%
Köder et al. (2024)	Multinational	Second-language learning	Y	Mixed methods, survey	Self-identified	Adults with ADHD	59	68%
Kyaga et al. (2013)	Sweden	Creativity	Y	Quant; retrospective analysis	Verified diagnosis	Patient registry data	1,173,763	34%
Lasky et al. (2016)	USA	General	N	Qual; interviews	Verified diagnosis	Young adults diagnosed with ADHD as children	125	24%
Lassinantti (2014)	Norway	General	Y	Qual; interviews	Self-reported diagnosis	Women with ADHD	16	100%
Lee et al. (2020)	South Korea	General	Y	Qual; content analysis	Other^a^	Famous/historical figures	131	11%
Leffers (1997)	USA	General	N	Qual; interviews, content analysis, observation	Self-reported diagnosis	Adults with ADD (diagnosed as adults)	41	59%
Limsuwan and Jullagate (2018)	Thailand	General	Y	Qual; in-depth interviews	Verified diagnosis	Adults with ADHD	9	33%
Mahdi et al. (2017)	Multinational	General	Y	Qual; interviews and focus groups	Self-reported diagnosis	Children and adults with ADHD, family members, professional caregivers	76	50% (adults with ADHD)
Mahdi et al. (2018)	Multinational	General	Y	Qual; interviews, content analysis	Verified diagnosis	Clinicians and clinical researchers	119	66%
Marchetta et al. (2008)	Netherlands	Executive functioning	N	Quant; quasi-exp	Self-reported diagnosis	Adults	212	25%–47%
McBride et al. (2021)	USA	Creativity	Y	Quant; within-subjects^e^	Self-reported diagnosis	Adults with ADHD	17	59%
Milthorp (2003)	Canada	Creativity	Y	Qual; dialogic interviewing	Self-reported diagnosis	Adult with ADHD	1	100%
Murray (2018)	USA	General	N	Qual; heuristic self-search inquiry	Self-reported diagnosis	Adult with ADHD and dyslexia	1	100%
Muszynska and Ossmy (2025)	UK	Problem solving	Y	Quant; quasi-exp	Screened (CAARS)	Adults	70	41%
Nash-Luckenbach (2020)	USA	General	N	Qual; interviews	Self-reported diagnosis	University students with ADHD	10	40%
Nixdorff (2008)	USA	Entrepreneurship	Y	Qual; interviews	Self-identified	Early-stage entrepreneurs	30	60%
Nordby et al. (2023)	Norway	General	Y	Qual; survey	Self-reported diagnosis	Adults with ADHD (diagnosed, help-seeking)	50	88%
Ottero (2016)	USA	General	N	Qual; ethnographic interviews	Self-reported diagnosis	Young adults with ADHD	11	18%
Peltonen et al. (2020)	Multinational	Entrepreneurship	Y	Quant; retrospective analysis	Other^a^	Adults	NA	NA
Popovic (2011)	Canada	General	Y	Qual; interviews and focus groups	Self-reported diagnosis	Women with ADHD	4	100%
Rajah et al. (2021)	UK	Entrepreneurship	Y	Quant; retrospective analysis	Screened (CBS, RBS)^b^	Individuals born within a single week in April 1970	11,237	51% (first time point)
Rosetti and Mendez (2024)	Mexico	Creativity	Y	Quant; survey	Screened (ASRS screener)	Adults	2,990	NS
Rowe et al. (2021)	UK	General	Y	Qual; interviews	Self-reported diagnosis	NHS employees with ADHD	7	57%
Ruffner (2023)	USA	Entrepreneurship	Y	Quant; survey	Screened (ASRS screener)	Graduate students	502	47%
Saldanha et al. (2022)	UK	Creativity	Y	Quant; survey	Screened (not stated)	Adults with joint hypermobility	104	NS
Sam et al. (2020)	USA	Creativity	Y	Quant; within-subjects^e^	Self-reported diagnosis	Adults	17	NS
Schäfer and Kraneburg (2015)	Germany	Justice sensitivity	Y	Quant; quasi-exp (matched samples)	Self-identified	Adults	50	44%–78%
Schecklmann et al. (2008)	Germany	Verbal fluency	N	Quant; quasi-exp	Verified diagnosis	Adults	28	36%–43%
Schippers et al. (2022)	Netherlands	General	Y	Mixed methods; survey	Screened (ASRS-18)	Adults with ADHD	206	63%
Schippers et al. (2024)	UK	General	Y	Quant; survey	Self-reported diagnosis	General adult population	694	50%
Schippers et al. (2024)	Netherlands	Sensory processing sensitivity	Y	Quant; survey	Self-reported diagnosis^f^	Adults	496	NS
Schott (2013)	USA	General	N	Qual; structured interviews	Self-reported diagnosis	Adults with ADHD	31	55%
Schreuer and Dorot (2017)	Israel	General	N	Qual; interviews	Verified diagnosis	Women with ADHD (employed)	11	100%
Schrevel et al. (2016)	Netherlands	General	Y	Qual; focus groups	Self-reported diagnosis	Adults with ADHD or ADD (primary diagnosis)	52	54%
Sedgwick et al. (2019)	UK	General	Y	Qual; interviews	Self-reported diagnosis	Successful adults with ADHD	6	0%
Sklar (2013)	South Africa	Hyperfocus	Y	Quant; quasi-exp	Screened (ASRS screener)	Adults	10	40%
Sônego et al. (2021)	Brazil	Entrepreneurship	Y	Quant; survey	Screened (ASRS screener)	Applicants for a career development course for entrepreneurs	259	35%
Steele et al. (2021)	Israel	Creativity	Y	Quant; survey	Screened (ASRS screener)	Adults (employed)	258	51%
Stenner et al. (2019)	UK	General	N	Qual; interviews	Self-identified	Women with ADHD (formally or self-diagnosed)	16	100%
Stolte et al. (2022)	Netherlands	Creativity	Y	Quant; quasi-exp, case-control	Screened (ASRS-18)	Adults	621	59%
Swan (2021)	USA	Hyperfocus	Y	Quant; scale development^a^	NA	Clinicians/researchers with relevant expertise	10	50%
Tal and Goodman (2025)	Israel	General	N	Qual; fieldwork	Self-reported diagnosis	Adults with ADHD (diagnosed in midlife)	36	56%
Taylor et al. (2020)	USA	Creativity	Y	Quant; quasi-exp	Screened (BADDS)	University students (engineering)	57	35%
Taylor et al. (2025)	UK	Creativity	Y	Quant; quasi-exp	Self-identified; screened (ASRS-18)	Autistic and non-autistic adults	352	49%
Toner et al. (2006)	Australia	General	N	Qual; interviews	Self-reported diagnosis	Adults with ADHD	10	0%
Tucha et al. (2011)	Germany	Creativity	Y	Quant; quasi-exp, within-subjects	Screened (CSS)	Adults	332	32%–42%
Verheul et al. (2016)	Multinational	Entrepreneurship	Y	Quant; survey	Screened (ASRS-18)	Swedish twin cohort population; Dutch student survey population	38,483	56%
Watters et al. (2018)	Ireland	General	N	Qual; interviews	Verified diagnosis	Adults with ADHD (outpatients)	11	18%
Westwater (2012)	USA	General	N	Qual; interviews	Self-reported diagnosis	Adults with ADHD (diagnosed and treated as children)	8	63%
White (2020)	USA	Creativity	Y	Quant; quasi-exp	Self-reported diagnosis; screened (CSS, CAARS)	University students	52	50%
White and Shah (2006)	USA	Creativity	Y	Quant; quasi-exp	Self-reported diagnosis; screened (CSS, CAARS)	University students	90	51%–53%
White and Shah (2011)	USA	Creativity	Y	Quant; quasi-exp	Self-reported diagnosis; screened (CSS, CAARS)	University students	60	48%
White and Shah (2016)	USA	Creativity	Y	Quant; quasi-exp	Self-reported diagnosis; screened (CSS, Childhood Symptoms Scale, BAADS)	University students	60	50%–60%
Wiklund et al. (2017)	USA	Entrepreneurship	Y	Quant; survey	Screened (ASRS screener)	MBA alumni	559	33%
Wilmshurst et al. (2011)	USA	General	N	Quant; quasi-exp	Self-reported diagnosis; screened (CSS, CAARS, CPT-II)	University students	36	56%
Wismans et al. (2020)	France	Entrepreneurship	Y	Quant; secondary data analysis	Screened (ASRS screener)	Small business owners	802	20%
Wright (2011)	USA	General	Y	Qual; interviews	Self-reported diagnosis	Adults with ADHD	10	60%
Yokell (2023)	USA	General	N	Qual; structured interviews	Self-reported diagnosis	Adults with ADHD (diagnosed)	15	NS
S. Young (2005)	UK	General	N	Quant; quasi-exp	Self-reported diagnosis	Adults	78	20%–38%
Z. Young et al. (2019)	UK	General	N	Qual; interviews	Verified diagnosis	Adults with ADHD (diagnosed in adulthood)	12	42%
Yu (2018)	USA	Entrepreneurship	Y	Quant; survey	Screened (ASRS screener)	MBA alumni	545	33%
Zabelina et al. (2014)	USA	Creativity	Y	Quant; quasi-exp	Screened (ASRS-18)	Adults	100	67%
Zaghi et al. (2016)	USA	Creativity	Y	Quant; survey	Screened (BADDS)	University students (engineering)	54	33%

*Note*. All quantitative studies utilised at least one comparison group unless otherwise stated; ADD = attention deficit disorder; ADHD = attention deficit hyperactivity disorder; ADHD-RS = ADHD Rating Scale (*n* = 1; DuPaul, 1998); ASRS-5 = Adult ADHD Self-Report Scale, DSM-5 version (*n* = 1;Ustun et al., 2017); ASRS-18 = Adult ADHD Self-Report Scale, 18-item version (*n* = 12; Kessler et al., 2005); ASRS screener = Adult ADHD Self-Report Scale, 6-item version (*n* = 11; Kessler et al., 2005); auADHD = autistic ADHD; BAADS = Brown Attention Deficit Disorder Scale – adult version (*n* = 2; Brown, 1996); CAARS = Conners Adult ADHD Rating Scales (*n* = 5; Conners et al., 1999); CBS = Childhood Behaviour Scale (*n* = 1; Ladd & Profilet, 1996); CPT-II = Conners’ Continuous Performance Test II (*n* = 1; Conners & MHS Staff, 2000); CSS = Current Symptoms Scale (*n* = 5); LD = learning disability; MBA = master of business administration; NHS = National Health Service (UK); NA = not applicable; NS = not stated; Qual = qualitative; Quant = quantitative; Quasi-exp = quasi-experimental; RBS = Rutter Behaviour Scale (*n* = 1; Elander & Rutter, 1996); WURS = Wender Utah Rating Scale (*n* = 2; Ward et al., 1993).

a Other: participants reported that their parents had ADHD (Belo-Tomic et al., 2021); retrospective potential diagnoses of celebrities (Lee et al., 2020); population-level methylphenidate use (Peltonen et al., 2020).

b Included subclinical symptoms (Boot et al., 2017) or “ADHD-like symptoms” (Rajah et al., 2021).

c Reported “confirmed diagnosis” but not stated how confirmed.

d No comparison group.

e Both within-subjects studies involved participants with ADHD and compared results when they had versus had not taken methylphenidate.

f Or in the process of diagnosis.

Many studies (58/125, 46%) included participants who reported that they had been diagnosed with ADHD by a healthcare professional; 11 of these involved additional measures (questionnaire, 8; confirmation of diagnosis or characteristics 2; questions about diagnosing clinician, symptoms, and functioning, 1), one included participants in the process of seeking a diagnosis, and one allowed either self-report of diagnosis or characteristics reported on a questionnaire. Twenty-nine studies used questionnaires to screen for ADHD, with one examining subclinical ADHD and another investigating “ADHD-like symptoms.” In 16 studies, participants were included if they self-identified as having ADHD, or if they reported having ADHD but the study did not specify whether they had a diagnosis; three of these used additional measures of ADHD (questionnaires, 2; asked to describe childhood challenges, 1). A minority of studies (17/125. 14%) verified participants’ diagnoses by sighting diagnostic reports, only accepting referrals from diagnosing healthcare professionals, or conducting diagnostic interviews. Three studies used other criteria (participants’ report of parents’ ADHD status, population-level use of methylphenidate, retrospective possible diagnosis of celebrities), and the participants of two studies were healthcare professionals.

Apart from two studies of participants with clinical or research expertise in working with ADHD (who did not have ADHD themselves), study participants included adults with ADHD (or ADHD characteristics). Four studies also included other relevant participants (e.g., parents of children with ADHD), and one study included child and adult participants. Some studies investigated specific populations, such as “successful” (defined in different ways, but generally relating to employment and income) adults with ADHD (*n* = 5) or women with ADHD (*n* = 9). Further characteristics of the included studies are shown in Table 1, and more detailed characteristics, including strengths mentioned in each study, are shown in Supplemental File 3.

### Study Topics and Strengths Mentioned

Basic qualitative analysis grouped strengths into 10 categories (Table 2; see also Supplemental File 3). One category, *interest-based attention* was adapted from previous research (Bertilsdotter Rosqvist et al., 2023a); here, this was conceptualised as the positive aspects of the diagnostic category of inattention. Similarly, *energy* and *adaptive risk-taking* can be seen as the positive sides of hyperactivity and impulsivity, respectively. These terms were chosen instead of *intensity* (Bertilsdotter Rosqvist et al., 2023a) to match diagnostic criteria more closely.

**Table 2.** Frequency of Strengths Mentioned in Included Studies, Grouped by Whether Strengths Were Identified A-Priori or Post-Hoc, and Methodology.

Strength	A-priori study of strengths			Post-hoc identification of strengths			Total	Examples
	Qualitative	Quantitative	Mixed methods	Qualitative	Quantitative	Mixed methods		
Creativity	27	31	3	17	4	0	82	Artistic ability; divergent thinking
Interest-based attention	25	11	2	12	2	0	52	Hyperfocus; seeing details others miss
Prosocial attributes	21	3	2	10	1	2	39	Empathy; being entertaining to others
Energy	22	0	1	10	0	0	33	High energy, productivity
Adaptive risk-taking	10	4	1	3	1	0	19	Courage; spontaneity; moderate risk-taking
Entrepreneurship	2	8	0	2	2	0	14	Entrepreneurial intention; self-employment
Resilience	10	2	0	3	0	1	16	Resilience; persistence
Flexibility	6	3	1	2	1	1	14	Adaptability; flexibility
Uniqueness	7	2	0	7	0	0	16	Individuality; authenticity
Other	7	3	1	7	6	0	24	Verbal fluency; spatial ability

*Note*. More detailed information on strengths mentioned in included studies is provided in Supplemental File 3.

Most studies (*n* = 68; 54%) were general in nature, covering multiple strengths or topics. Other common topics were specific to a particular strength (e.g., creativity; entrepreneurship). Eighty-eight studies (70%) had an a-priori aim, research question, or research method (e.g., interview or survey question) about ADHD-related strengths (quantitative, *n* = 49; qualitative, *n* = 36; mixed methods, *n* = 3).

Conversely, 37 (30%) did not set out to study strengths, including 27 studies investigating experiences of ADHD from a neutral phenomenological standpoint. In these studies, which were predominantly qualitative (*n* = 23) or mixed methods (*n* = 1), participants described experiences of ADHD-related strengths unprompted by researchers. The remaining 10 studies, which were predominantly quantitative (*n* = 7) had explicitly deficit-based hypotheses about ADHD, and authors speculated on potential ADHD-related strengths after obtaining results that were inconsistent with their hypotheses.

In some cases, the topic of the study and the strengths mentioned were the same; this was often the case for creativity and entrepreneurship. Other categories (e.g., flexibility, uniqueness) and strengths mentioned were not the a-priori subject of any study, but were mentioned by participants in general studies, or as potential explanations for results in studies with specific topics.

#### Creativity

Creativity was the most frequently studied and mentioned category across qualitative and quantitative studies (Table 2). Whereas 27 studies (22%) focussed on creativity in relation to ADHD, it was also frequently mentioned in general and other studies, resulting in 82 mentions overall (66%). This category encompassed artistic ability and creative professions, problem solving (including convergent and divergent thinking), and learned creativity, or creative use of strengths and strategies to overcome challenges (Gerber, 2001). Quantitative studies used a range of measures of creativity, including the Creative Achievement Questionnaire (Carson et al., 2005), the Alternate Uses Task (Guilford, 1967), the Remote Associates Test (Mednick, 1962), Torrance Tests of Creative Thinking (Torrance, n.d.), and many other self-report measures and creative tasks. Three studies, all of which investigated the effect of methylphenidate on creativity among people with ADHD, used measures of verbal fluency as part of their assessment of divergent thinking (McBride et al., 2021; Sam et al., 2020; Tucha et al., 2011).

#### Interest-Based Attention

Whereas 11 studies (9%) set out to investigate topics related to interest-based attention, 52 studies (42%) mentioned strengths in this category. Most mentions occurred in qualitative research (Table 2). This category included hyperfocus; curiosity, including responding positively to novelty and reward; multitasking or rapid task-switching; heightened sensitivity to environmental stimuli (sensory sensitivity, reduced inattentional blindness); performing better under pressure; fast or quick thinking; and positive aspects of mind wandering. Several responses directly referenced interest, such as improved focus or productivity when engaged in a topic. Quantitative studies of interest-based attention used a range of outcome measures: the Adult Hyperfocus Questionnaire (Hupfeld et al., 2019) or a subscale thereof; the Sensory Profile (Dunn, 1999); electroencephalogram (EEG); or behavioural tasks such as a continuous performance task (Mueller & Piper, 2014), an online foraging game (Barack et al., 2024), or an inattentional blindness task comprising two continuous performance tasks (Berger & Cassuto, 2014; Simons, 2010).

#### Prosocial Attributes

Three studies investigated prosocial attributes directly, while 39 (31%) mentioned traits such as empathy or compassion; being funny and entertaining; justice sensitivity; communication skills; and willingness to help others. Prosocial attributes were mentioned more frequently in qualitative studies than quantitative (Table 2). The two quantitative studies investigating prosocial attributes a-priori both used multiple measures of their chosen topics: Engelthaler (2021) adapted several scales on social media usage and observed participants’ Instagram accounts, whereas Schäfer and Kraneburg (2015) observed participants’ behaviour in a justice game and used an existing justice sensitivity questionnaire (Schmitt et al., 2009).

#### Energy

Despite no studies examining energy specifically, 33 (26%) mentioned related traits, predominantly having high energy, drive, enthusiasm, and passion. All of these studies were qualitative, with the exception of one mixed-methods study whose quantitative methods did not pertain to energy (Köder et al., 2024). Participants often directly related these characteristics to increased productivity and efficiency.

#### Adaptive Risk-Taking

Adaptive risk-taking was not the topic of any study, although it was frequently discussed in the context of entrepreneurship. Nineteen studies (15%), predominantly qualitative (Table 2) referenced qualities such as adventurousness, bravery, courage, and fearlessness; moderate risk-taking; and spontaneity.

#### Entrepreneurship

Fourteen studies (11%) touched on entrepreneurship; most of these were quantitative (Table 2). Of these, 11 studies (9%) specifically examined entrepreneurship (variously defined as self-employment, or entrepreneurial intent, cognition or behaviour), and the remaining three (2%) mentioned this category unprompted. Some of these studies were published in business or economics journals (Dimic & Orlov, 2015; Peltonen et al., 2020; Rajah et al., 2021; Wiklund et al., 2016); one of these was a longitudinal study with 11,237 participants (Rajah et al., 2021), while an epidemiological study included two samples of 25,364 and 13,119 participants (Verheul et al., 2016). Quantitative studies used a range of measures to study entrepreneurship, including behavioural measures (e.g., business ownership, or measures of entrepreneurial performance such as business profits) and self-report measures (Bolton & Lane, 2012; Caird, 1988; Covin & Slevin, 1988; Liñán & Chen, 2009; Zhao et al., 2005).

#### Resilience and Flexibility

Resilience was the topic of one study, and it was covered by 16 (13%), which also used terms such as persistence, and determination. Despite no studies examining flexibility directly, 14 studies (11%) mentioned either adaptability or flexibility (Katabi et al., 2023). For both categories, most studies were qualitative (Table 2).

#### Uniqueness and Other Characteristics

Although uniqueness was not the topic of any included study, 16 studies (13%) mentioned traits such as individuality, authenticity, autonomy, and being an interesting person. Most of these studies were qualitative (Table 2). Characteristics with fewer than 10 mentions were collated into “other” (*n* = 24; 19%), which included hands-on learning and spatial ability, verbal fluency, and vocabulary size.

## Discussion

This scoping review aimed to determine the extent of empirical qualitative and quantitative research about strengths that may be related to ADHD in adults. The study included 125 empirical studies and comprised peer-reviewed articles, dissertations, and grey literature. While most studies (70%) set out to consider ADHD strengths a-priori, the remainder did not. Strengths mentioned ranged from personal attributes and cognitive skills to positive aspects of ADHD itself. The review found the most mentioned strength was creativity (66%); while others pertained to potential positive aspects of inattention (42%), hyperactivity (26%), or impulsivity (15%); prosocial attributes (31%); resilience (13%); uniqueness (13%); entrepreneurship (11%); flexibility (11%); as well as other characteristics (19%). Most potential strengths were more frequently mentioned in qualitative studies than quantitative, except for creativity, which has been studied using a range of methodologies, and entrepreneurship, which was more commonly studied using quantitative methodologies.

Participants in many included studies reframed core diagnostic criteria of ADHD as potential strengths. In this conceptualisation, ADHD-related strengths may be more than side benefits or silver linings of a neurobiological difference involving many challenges; rather, they are potentially inherent to the experience of ADHD. In this review, positive aspects of inattention were collectively termed *interest-based attention* (adapted from Bertilsdotter Rosqvist et al., 2023a), hyperactivity was referred to as *energy*, and impulsivity was conceptualised as *adaptive risk-taking*. Although this review did not consider whether strengths mentioned were empirically related to ADHD, it is important to note that some, if not all, ADHD characteristics may be strengths in some contexts and challenges in others (Bertilsdotter Rosqvist et al., 2023a). For example, impulsivity can be associated with dangerous risk-taking behaviours and resultant negative outcomes, such as unwanted pregnancy and death (Faraone et al., 2021). Conversely, in contexts where taking risks is requisite for success, risk-taking behaviours may be appropriate and therefore adaptive, as seen in one study examining predictors of entrepreneurial tendency, which found a key role for moderate risk-taking (Dimic & Orlov, 2015). Interestingly, several studies on ADHD and entrepreneurship were conducted outside of mental health contexts (Dimic & Orlov, 2015; Peltonen et al., 2020; Rajah et al., 2021; Wiklund et al., 2016). While it is understandable that mental health clinicians and researchers tend to focus on challenges, considering strengths associated with this may offer useful paradigm shifts for mental health research. Instead of assuming that all ADHD characteristics must be deficits, or alternatively, assuming all ADHD characteristics are strengths, it may be helpful to consider the relationships between these traits. If some traits may be strengths in some contexts and challenges in others, as outlined by Bertilsdotter Rosqvist et al. (2023a), people with ADHD may sometimes be better served by changing their environment (or by these environments striving to become more neurodiversity-affirming), rather than trying to change themselves. Although warranting further study, such concepts could begin to be incorporated into clinical practice guidelines—both on individual levels, such as helping people with ADHD find suitable employment; and on structural levels, such as institution-level advocacy programmes.

Such perspectives offer an opportunity to consider the overall state of ADHD research. The studies included in this review—the largest synthesis of studies on ADHD-related strengths to date—represent a minority in the field when compared to studies that examine ADHD deficits (Chaulagain et al., 2023). Indeed, 83% of the 132 studies removed at the full-text screening stage of this review were excluded because they did not consider ADHD-related strengths. This mirrors previous reviews where no strengths were found despite efforts to include this information (de Schipper et al., 2015), and where strengths were mentioned in just three (1.3%) of 231 reviews in a recent meta-review (Chaulagain et al., 2023). While support for ADHD challenges is important, this preponderance of deficit-based research may impede researchers and clinicians from considering ADHD strengths, wellbeing, and growth (Champ et al., 2021).

A useful starting point in such work may be further scrutiny of the studies included in this review, many of which suggest further depth and richness of ADHD characteristics, with corresponding avenues for research. For example, interest-based attention included the concept of hyperfocus, a state of intense and prolonged concentration that is associated with ADHD but not included in diagnostic criteria (Ashinoff & Abu-Akel, 2021). Hyperfocus has been studied through a deficit-based lens, as it can lead to poorer academic and occupational outcomes as well as physical discomfort (e.g., Ayers-Glassey & MacIntyre, 2021; Ayers-Glassey & Smilek, 2024; Ishii et al., 2023). More broadly, attentional challenges are often studied using computer-based tasks that require participants to monitor for infrequent, tedious stimuli; unsurprisingly, people with ADHD respond more slowly and inconsistently in such tests (for review, see Ging-Jehli et al., 2021). Conversely, studies included in this review identified hyperfocus as a strength associated with ADHD, citing its usefulness when harnessed for valued tasks relevant to the person’s interests (e.g., Hupfeld et al., 2019). Taken together, this body of research suggests that attention in ADHD can be variable, and even a strength at times (Champ et al., 2023). Further research to better understand the conditions that lead to positive hyperfocus may prove fruitful. Very little appears to be empirically known about what people with ADHD are attending to, rather than what they are not. Similarly, motivation in the context of ADHD, categorised in this review as part of *energy*, has only been studied through a deficit-based lens in children and adolescents (Smith & Langberg, 2018), as well as adults (Capps et al., 2023). However, Capps et al. (2023) found that ADHD traits moderated the effect of mastery-based motivation on grade point average among university students, such that participants with moderate-to-high levels of ADHD traits who also had high mastery goal orientation reported higher academic achievements. Similar patterns have been seen in adolescents (Smith et al., 2020). While theoretical models relating to ADHD and motivation have been proposed that may relate to interest-based attention (Champ et al., 2023; DeWitt, 2020), they are yet to be studied empirically.

Researchers have also rightly raised concerns about methodological issues in the field (Ashinoff & Abu-Akel, 2021; Hoogman et al., 2020; Paek et al., 2016). Creativity, the most frequently mentioned strength in this review (69%), referred to a range of skills, from artistic tendencies to problem-solving abilities (Boot et al., 2020; Kyaga et al., 2013; White, 2020). For example, Boot et al. (2020) focussed on problem-solving, employing an alternative uses task and self-report measures of creativity; Kyaga et al. (2013) focussed on choice of profession; whereas White (2020) assessed the originality of participants’ drawings based on a prompt to create an alien fruit. Earlier reviews have noted that this heterogeneity hampers comparison between studies, eroding their usefulness and empirical quality (Hoogman et al., 2020; Paek et al., 2016), and measurement tools for creativity have long been criticised for their poor psychometric properties (Thys et al., 2014). This area of research requires further rigour if any relationship between creativity and ADHD is to be definitively established. Nonetheless, the high proportion of general studies mentioning creativity (*n* = 59/125; 47%) is a potential convergent stream of evidence.

While the heterogeneity of creativity research may be a limitation in research contexts, the variety and even uncertainty may be useful for clinicians who are exploring strengths with their clients. The different aspects of creativity, from artistic pursuits to unorthodox problem solving, may appeal to different individuals with ADHD. The inclusion of learned creativity (Gerber, 2001) is also of particular interest, as this may demonstrate a specific application of creative and problem-solving strengths to manage challenges (Abramowicz, 1998; Davis, 2014). This specific form of creativity may even relate to the concepts of resilience and flexibility, although such links have not been researched. Research into ADHD and resilience has focussed on protective factors relating to improved quality of life and transition to adult care for adolescents, and better prognoses for adults with ADHD who experience depression (Oddo et al., 2018; Schei et al., 2015, 2018). Conversely, the present review conceptualised resilience as a strength arising from ADHD, or lived experience thereof, including its challenges. Conceptually, flexibility may relate to resilience as both are required to manage challenges (Dean et al., 2018). However, flexibility may also relate to adaptive risk-taking; research (not specific to ADHD) has shown that sensation seeking, a concept related to adaptive risk-taking, is associated with active coping strategies for pain management (Meredith et al., 2015). The potential links between these concepts and creativity illustrate the overlapping and underexplored nature of ADHD-related strengths.

These potential differences in creativity, resilience, and other categories outside those conceptualised as positives of core ADHD traits, point to the value of further study in these areas. If these strengths present or are developed differently in people with ADHD than their peers, this may point to subtle characteristics that are still worthy of nurturance and celebration, in line with strengths-based methods and the neurodiversity paradigm (Dwyer, 2022; Flückiger et al., 2023). One relevant example is the specific nature of prosocial skills among people with ADHD. In qualitative studies, teachers with ADHD described experiences of increased empathy towards their students due to their own challenges (Brock, 2008), and older women with ADHD related lifelong experiences of advocating for vulnerable people (Henry & Jones, 2011). In their study of justice sensitivity in ADHD, Schäfer and Kraneburg (2015) reflected that justice sensitivity may arise in ADHD as a way to compensate for challenges with following social norms. A similar study in adolescents, focussing on prosocial risk-taking, found that participants with ADHD were more likely to engage in prosocial risk-taking than their peers. These findings suggest an interaction between the innate characteristics of ADHD, and the experiences of people who have it. However, this area merits further research; for example, although described as a pilot study, Schäfer and Kraneburg’s (2015) justice sensitivity study is yet to be replicated, 10 years after its publication.

Further, some studies (14%) included in this review referenced the quality of uniqueness, or being an interesting or unusual person. While this concept has not been researched specifically, and may be difficult to operationalise, it may be of interest to people with lived experience of ADHD and their clinicians due to its potential links with the neurodiversity framework. In line with other pride movements, early results suggest that positive autistic identities are beneficial for mental wellbeing and self-esteem (Barnhart, 2016; Chapman & Botha, 2023; Cooper et al., 2017). As ADHD is associated with low self-esteem (Newark et al., 2016) and internalised stigma (Masuch et al., 2019), it stands to reason that thinking of oneself as unique may be protective. However, this concept requires further research. Similarly, considering even the possibility of ADHD-related strengths more broadly, and specific to each individual, may improve self-esteem and quality of life for people with ADHD. The utility of this concept could be explored further by clinical guidelines developers.

### Future Directions

While this review outlines the extent of research into ADHD-related strengths in adults, it has also identified many directions for future research. Some strengths identified here have been studied extensively, yet research limitations persist in some areas, such as creativity, due to methodological concerns. Other potential strengths have never been studied in depth (e.g., energy, adaptive risk-taking), or have only or predominantly been studied as deficits (e.g., prosocial skills, hyperfocus, motivation). The categories best represented here by qualitative research would benefit from quantitative approaches, using these in-depth studies for hypothesis generation. Conversely, entrepreneurship was rarely studied qualitatively; in-depth qualitative methodologies could yield rich insights into this intriguing topic. Further, even among strengths that have been examined in multiple quantitative studies, a variety of measures have been used, from computer games and tasks to self-report measures. Although beyond the scope of this review, further scrutiny of the psychometric properties of these measures would likely improve the quality of research in this field.

It is unclear whether some of these strengths have a neurological basis, or arise as learned strategies to manage the challenges of living with ADHD in a world not designed for the full spectrum of neurodiversity (e.g., resilience). Each of these aspects could be studied in high-quality research co-designed by people with lived experience to better inform our understanding of ADHD and develop related clinical applications. Such approaches are an important first step in addressing epistemic injustice (Byskov, 2021; Eagly & Riger, 2014; Teo, 2022) and ensuring that all potential ADHD strengths are understood, harnessed, and celebrated.

Additionally, a similar review could be conducted into ADHD-related strengths in children and adolescents, and a meta-synthesis of the qualitative studies would provide greater depth and further insights into this topic. Although reviews examining childhood and adolescent experiences of ADHD yielded strengths mentioned in this review (Eccleston et al., 2019; Wong et al., 2018), it is possible that some ADHD strengths are present even in early life, whereas others develop over the course of the lifespan.

### Limitations

Despite the extensive findings of this review, it has limitations. As a scoping review, it sought to map the extent of the field, and was not geared to examine causation, correlation, or the validity of any of the constructs described. Additionally, as the population, concept and concept were chosen to favour breadth, in line with the purpose of scoping reviews, some “false positives”—participants who do not meet diagnostic criteria for ADHD, or strengths that are not related to ADHD—may be represented here. Only a small proportion of studies verified participants’ diagnoses, with most relying on self-report or screening measures as inclusion criteria. Although all studies were empirical, some are included based on their conclusions, rather than their results. Such studies were included for the purpose of hypothesis generation, and further research must be conducted before definitive conclusions can be drawn.

It should also be stressed that the results were categorised using basic qualitative analysis, so these themes should not be considered definitive. Some categories may be too broad and others too narrow, which may provide yet another avenue for further research. The ICF core set identified for ADHD may provide a useful framework for a more deductive approach (Bölte et al., 2021).

Most articles were published in English and originated from North America and Europe. It is unclear whether studies from other regions have been published but not indexed by the databases searched here, or whether the evidence base in other regions is still developing. Further, as sociodemographic factors were not extracted (except for age, gender, and any specific category noted by individual studies), it is unknown how representative these studies are, even within their countries of origin. Such demographic considerations would also benefit from further research.

These limitations all highlight the potential of a review such as this to contribute to epistemic injustice if not contextualised adequately. Even as it has attempted to capture breadth, restricting results to empirical evidence and specific demographics privileges specific ways of knowing at the expense of others. This is exacerbated by the state of research more broadly, as seen by the insufficient representation from the Global South. This review should be seen as a map of part of a body of knowledge, with many waters still waiting to be charted by research.

## Conclusions

This scoping review sought to investigate the breadth of empirical research about strengths that may be related to ADHD in adults. While a variety of studies on a range of strengths were included, they represent a small proportion of ADHD research, and many gaps and questions remain. All core ADHD characteristics are perceived as strengths by some people in some contexts, and several other strengths were identified across studies. Further research, followed by updated clinical practice guidelines, relating to ADHD strengths are needed, so that mental health professionals can support people with ADHD through their challenges while helping them develop and utilise their strengths in contexts where they are most likely to flourish.

## Supplemental Material

sj-docx-1-jad-10.1177_10870547261425737 – Supplemental material for Attention Deficit/Hyperactivity Disorder (ADHD)-Related Strengths in Adults: A Scoping Review Supplemental material, sj-docx-1-jad-10.1177_10870547261425737 for Attention Deficit/Hyperactivity Disorder (ADHD)-Related Strengths in Adults: A Scoping Review by Rivqa B. Rafael, Hongwei Jia, Melissa Rouel, Bethany M. Wootton and Deborah Mitchison in Journal of Attention Disorders

sj-docx-2-jad-10.1177_10870547261425737 – Supplemental material for Attention Deficit/Hyperactivity Disorder (ADHD)-Related Strengths in Adults: A Scoping Review Supplemental material, sj-docx-2-jad-10.1177_10870547261425737 for Attention Deficit/Hyperactivity Disorder (ADHD)-Related Strengths in Adults: A Scoping Review by Rivqa B. Rafael, Hongwei Jia, Melissa Rouel, Bethany M. Wootton and Deborah Mitchison in Journal of Attention Disorders

sj-xlsx-3-jad-10.1177_10870547261425737 – Supplemental material for Attention Deficit/Hyperactivity Disorder (ADHD)-Related Strengths in Adults: A Scoping Review Supplemental material, sj-xlsx-3-jad-10.1177_10870547261425737 for Attention Deficit/Hyperactivity Disorder (ADHD)-Related Strengths in Adults: A Scoping Review by Rivqa B. Rafael, Hongwei Jia, Melissa Rouel, Bethany M. Wootton and Deborah Mitchison in Journal of Attention Disorders
