# Supplementary material for: Attention Deficit/Hyperactivity Disorder (ADHD)-Related Strengths in Adults: A Scoping Review
Source: J Atten Disord. 2026 Mar 26;30(10):1207–32. doi: 10.1177/10870547261425737 (PMC13538910; doi:10.1177/10870547261425737)
Supplement: Supplementary file 1 [file sj-docx-1-jad-10.1177_10870547261425737.docx]

Supplementary File 1: Search Strategy

Search strings

Embase

1. attention deficit hyperactivity disorder/

2. adhd.mp. [mp=title, abstract, heading word, drug trade name, original title, device manufacturer, drug manufacturer, device trade name, keyword heading word, floating subheading word, candidate term word]

3. attention deficit hyperactivity disorder.mp. [mp=title, abstract, heading word, drug trade name, original title, device manufacturer, drug manufacturer, device trade name, keyword heading word, floating subheading word, candidate term word]

4. attention deficit disorder.mp. [mp=title, abstract, heading word, drug trade name, original title, device manufacturer, drug manufacturer, device trade name, keyword heading word, floating subheading word, candidate term word]

5. 1 or 2 or 3 or 4

6. Adult/

7. adult*.mp. [mp=title, abstract, heading word, drug trade name, original title, device manufacturer, drug manufacturer, device trade name, keyword heading word, floating subheading word, candidate term word]

8. 6 or 7

9. 5 and 8

10. motivation/ or achievement/

11. strength*.mp. [mp=title, abstract, heading word, drug trade name, original title, device manufacturer, drug manufacturer, device trade name, keyword heading word, floating subheading word, candidate term word]

12. positives.mp. [mp=title, abstract, heading word, drug trade name, original title, device manufacturer, drug manufacturer, device trade name, keyword heading word, floating subheading word, candidate term word]

13. (positive adj1 aspect*).mp. [mp=title, abstract, heading word, drug trade name, original title, device manufacturer, drug manufacturer, device trade name, keyword heading word, floating subheading word, candidate term word]

14. (positive adj1 attribute*).mp. [mp=title, abstract, heading word, drug trade name, original title, device manufacturer, drug manufacturer, device trade name, keyword heading word, floating subheading word, candidate term word]

15. (positive adj1 trait*).mp. [mp=title, abstract, heading word, drug trade name, original title, device manufacturer, drug manufacturer, device trade name, keyword heading word, floating subheading word, candidate term word]

16. achievement*.mp. [mp=title, abstract, heading word, drug trade name, original title, device manufacturer, drug manufacturer, device trade name, keyword heading word, floating subheading word, candidate term word]

17. Psychology, Positive/

18. positive psychology.mp. [mp=title, abstract, heading word, drug trade name, original title, device manufacturer, drug manufacturer, device trade name, keyword heading word, floating subheading word, candidate term word]

19. motivat*.mp. [mp=title, abstract, heading word, drug trade name, original title, device manufacturer, drug manufacturer, device trade name, keyword heading word, floating subheading word, candidate term word]

20. passion*.mp. [mp=title, abstract, heading word, drug trade name, original title, device manufacturer, drug manufacturer, device trade name, keyword heading word, floating subheading word, candidate term word]

21. vigo?r.mp. [mp=title, abstract, heading word, drug trade name, original title, device manufacturer, drug manufacturer, device trade name, keyword heading word, floating subheading word, candidate term word]

22. enthus*.mp. [mp=title, abstract, heading word, drug trade name, original title, device manufacturer, drug manufacturer, device trade name, keyword heading word, floating subheading word, candidate term word]

23. creativity/ or empathy/

24. Problem Solving/

25. creativ*.mp. [mp=title, abstract, heading word, drug trade name, original title, device manufacturer, drug manufacturer, device trade name, keyword heading word, floating subheading word, candidate term word]

26. problem solving.mp. [mp=title, abstract, heading word, drug trade name, original title, device manufacturer, drug manufacturer, device trade name, keyword heading word, floating subheading word, candidate term word]

27. hyperfocus.mp. [mp=title, abstract, heading word, drug trade name, original title, device manufacturer, drug manufacturer, device trade name, keyword heading word, floating subheading word, candidate term word]

28. communication/ or exploratory behavior/ or multitasking behavior/

29. curios*.mp. [mp=title, abstract, heading word, drug trade name, original title, device manufacturer, drug manufacturer, device trade name, keyword heading word, floating subheading word, candidate term word]

30. multitask*.mp. [mp=title, abstract, heading word, drug trade name, original title, device manufacturer, drug manufacturer, device trade name, keyword heading word, floating subheading word, candidate term word]

31. altruism/ or cooperation/ or social competence/

32. imagination/ or intuition/

33. social justice.mp. or Social Justice/

34. advocat*.mp. [mp=title, abstract, heading word, drug trade name, original title, device manufacturer, drug manufacturer, device trade name, keyword heading word, floating subheading word, candidate term word]

35. communic*.mp. [mp=title, abstract, heading word, drug trade name, original title, device manufacturer, drug manufacturer, device trade name, keyword heading word, floating subheading word, candidate term word]

36. compassion*.mp. [mp=title, abstract, heading word, drug trade name, original title, device manufacturer, drug manufacturer, device trade name, keyword heading word, floating subheading word, candidate term word]

37. empath*.mp. [mp=title, abstract, heading word, drug trade name, original title, device manufacturer, drug manufacturer, device trade name, keyword heading word, floating subheading word, candidate term word]

38. interpersonal skill*.mp. [mp=title, abstract, heading word, drug trade name, original title, device manufacturer, drug manufacturer, device trade name, keyword heading word, floating subheading word, candidate term word]

39. intuiti*.mp. [mp=title, abstract, heading word, drug trade name, original title, device manufacturer, drug manufacturer, device trade name, keyword heading word, floating subheading word, candidate term word]

40. playful*.mp. [mp=title, abstract, heading word, drug trade name, original title, device manufacturer, drug manufacturer, device trade name, keyword heading word, floating subheading word, candidate term word]

41. adaptive behavior/

42. courage/ or hope/

43. psychological resilience/

44. brave*.mp. [mp=title, abstract, heading word, drug trade name, original title, device manufacturer, drug manufacturer, device trade name, keyword heading word, floating subheading word, candidate term word]

45. courage*.mp. [mp=title, abstract, heading word, drug trade name, original title, device manufacturer, drug manufacturer, device trade name, keyword heading word, floating subheading word, candidate term word]

46. entrepreneur*.mp. [mp=title, abstract, heading word, drug trade name, original title, device manufacturer, drug manufacturer, device trade name, keyword heading word, floating subheading word, candidate term word]

47. flexi*.mp. [mp=title, abstract, heading word, drug trade name, original title, device manufacturer, drug manufacturer, device trade name, keyword heading word, floating subheading word, candidate term word]

48. productiv*.mp. [mp=title, abstract, heading word, drug trade name, original title, device manufacturer, drug manufacturer, device trade name, keyword heading word, floating subheading word, candidate term word]

49. resilien*.mp. [mp=title, abstract, heading word, drug trade name, original title, device manufacturer, drug manufacturer, device trade name, keyword heading word, floating subheading word, candidate term word]

50. neurodivers*.mp. [mp=title, abstract, heading word, drug trade name, original title, device manufacturer, drug manufacturer, device trade name, keyword heading word, floating subheading word, candidate term word]

51. neurodiverg*.mp. [mp=title, abstract, heading word, drug trade name, original title, device manufacturer, drug manufacturer, device trade name, keyword heading word, floating subheading word, candidate term word]

52. neurodiversity/

53. entrepreneurship/

54. 11 or 12 or 13 or 14 or 15 or 16

55. 9 and 54

Medline

1. Attention Deficit Disorder with Hyperactivity/

2. adhd.mp. [mp=title, book title, abstract, original title, name of substance word, subject heading word, floating sub-heading word, keyword heading word, organism supplementary concept word, protocol supplementary concept word, rare disease supplementary concept word, unique identifier, synonyms, population supplementary concept word, anatomy supplementary concept word]

3. attention deficit hyperactivity disorder.mp. [mp=title, book title, abstract, original title, name of substance word, subject heading word, floating sub-heading word, keyword heading word, organism supplementary concept word, protocol supplementary concept word, rare disease supplementary concept word, unique identifier, synonyms, population supplementary concept word, anatomy supplementary concept word]

4. attention deficit disorder.mp. [mp=title, book title, abstract, original title, name of substance word, subject heading word, floating sub-heading word, keyword heading word, organism supplementary concept word, protocol supplementary concept word, rare disease supplementary concept word, unique identifier, synonyms, population supplementary concept word, anatomy supplementary concept word]

5. 1 or 2 or 3 or 4

6. Adult/

7. adult*.mp. [mp=title, book title, abstract, original title, name of substance word, subject heading word, floating sub-heading word, keyword heading word, organism supplementary concept word, protocol supplementary concept word, rare disease supplementary concept word, unique identifier, synonyms, population supplementary concept word, anatomy supplementary concept word]

8. 6 or 7

9. 5 and 8

10. motivation/ or achievement/ or aspirations, psychological/

11. strength*.mp. [mp=title, book title, abstract, original title, name of substance word, subject heading word, floating sub-heading word, keyword heading word, organism supplementary concept word, protocol supplementary concept word, rare disease supplementary concept word, unique identifier, synonyms, population supplementary concept word, anatomy supplementary concept word]

12. positives.mp. [mp=title, book title, abstract, original title, name of substance word, subject heading word, floating sub-heading word, keyword heading word, organism supplementary concept word, protocol supplementary concept word, rare disease supplementary concept word, unique identifier, synonyms, population supplementary concept word, anatomy supplementary concept word]

13. (positive adj1 aspect*).mp. [mp=title, book title, abstract, original title, name of substance word, subject heading word, floating sub-heading word, keyword heading word, organism supplementary concept word, protocol supplementary concept word, rare disease supplementary concept word, unique identifier, synonyms, population supplementary concept word, anatomy supplementary concept word]

14. (positive adj1 attribute*).mp. [mp=title, book title, abstract, original title, name of substance word, subject heading word, floating sub-heading word, keyword heading word, organism supplementary concept word, protocol supplementary concept word, rare disease supplementary concept word, unique identifier, synonyms, population supplementary concept word, anatomy supplementary concept word]

15. (positive adj1 trait*).mp. [mp=title, book title, abstract, original title, name of substance word, subject heading word, floating sub-heading word, keyword heading word, organism supplementary concept word, protocol supplementary concept word, rare disease supplementary concept word, unique identifier, synonyms, population supplementary concept word, anatomy supplementary concept word]

16. achievement*.mp. [mp=title, book title, abstract, original title, name of substance word, subject heading word, floating sub-heading word, keyword heading word, organism supplementary concept word, protocol supplementary concept word, rare disease supplementary concept word, unique identifier, synonyms, population supplementary concept word, anatomy supplementary concept word]

17. Psychology, Positive/

18. positive psychology.mp. [mp=title, book title, abstract, original title, name of substance word, subject heading word, floating sub-heading word, keyword heading word, organism supplementary concept word, protocol supplementary concept word, rare disease supplementary concept word, unique identifier, synonyms, population supplementary concept word, anatomy supplementary concept word]

19. motivat*.mp. [mp=title, book title, abstract, original title, name of substance word, subject heading word, floating sub-heading word, keyword heading word, organism supplementary concept word, protocol supplementary concept word, rare disease supplementary concept word, unique identifier, synonyms, population supplementary concept word, anatomy supplementary concept word]

20. passion*.mp. [mp=title, book title, abstract, original title, name of substance word, subject heading word, floating sub-heading word, keyword heading word, organism supplementary concept word, protocol supplementary concept word, rare disease supplementary concept word, unique identifier, synonyms, population supplementary concept word, anatomy supplementary concept word]

21. vigo?r.mp. [mp=title, book title, abstract, original title, name of substance word, subject heading word, floating sub-heading word, keyword heading word, organism supplementary concept word, protocol supplementary concept word, rare disease supplementary concept word, unique identifier, synonyms, population supplementary concept word, anatomy supplementary concept word]

22. enthus*.mp. [mp=title, book title, abstract, original title, name of substance word, subject heading word, floating sub-heading word, keyword heading word, organism supplementary concept word, protocol supplementary concept word, rare disease supplementary concept word, unique identifier, synonyms, population supplementary concept word, anatomy supplementary concept word]

23. creativity/ or empathy/

24. Problem Solving/

25. creativ*.mp. [mp=title, book title, abstract, original title, name of substance word, subject heading word, floating sub-heading word, keyword heading word, organism supplementary concept word, protocol supplementary concept word, rare disease supplementary concept word, unique identifier, synonyms, population supplementary concept word, anatomy supplementary concept word]

26. problem solving.mp. [mp=title, book title, abstract, original title, name of substance word, subject heading word, floating sub-heading word, keyword heading word, organism supplementary concept word, protocol supplementary concept word, rare disease supplementary concept word, unique identifier, synonyms, population supplementary concept word, anatomy supplementary concept word]

27. hyperfocus.mp. [mp=title, book title, abstract, original title, name of substance word, subject heading word, floating sub-heading word, keyword heading word, organism supplementary concept word, protocol supplementary concept word, rare disease supplementary concept word, unique identifier, synonyms, population supplementary concept word, anatomy supplementary concept word]

28. communication/ or exploratory behavior/ or multitasking behavior/

29. curios*.mp. [mp=title, book title, abstract, original title, name of substance word, subject heading word, floating sub-heading word, keyword heading word, organism supplementary concept word, protocol supplementary concept word, rare disease supplementary concept word, unique identifier, synonyms, population supplementary concept word, anatomy supplementary concept word]

30. multitask*.mp. [mp=title, book title, abstract, original title, name of substance word, subject heading word, floating sub-heading word, keyword heading word, organism supplementary concept word, protocol supplementary concept word, rare disease supplementary concept word, unique identifier, synonyms, population supplementary concept word, anatomy supplementary concept word]

31. altruism/ or cooperative behavior/ or helping behavior/ or social skills/

32. imagination/ or intuition/

33. social justice.mp. or Social Justice/

34. (wit and humor as topic).mp. [mp=title, book title, abstract, original title, name of substance word, subject heading word, floating sub-heading word, keyword heading word, organism supplementary concept word, protocol supplementary concept word, rare disease supplementary concept word, unique identifier, synonyms, population supplementary concept word, anatomy supplementary concept word]

35. advocat*.mp. [mp=title, book title, abstract, original title, name of substance word, subject heading word, floating sub-heading word, keyword heading word, organism supplementary concept word, protocol supplementary concept word, rare disease supplementary concept word, unique identifier, synonyms, population supplementary concept word, anatomy supplementary concept word]

36. communic*.mp. [mp=title, book title, abstract, original title, name of substance word, subject heading word, floating sub-heading word, keyword heading word, organism supplementary concept word, protocol supplementary concept word, rare disease supplementary concept word, unique identifier, synonyms, population supplementary concept word, anatomy supplementary concept word]

37. compassion*.mp. [mp=title, book title, abstract, original title, name of substance word, subject heading word, floating sub-heading word, keyword heading word, organism supplementary concept word, protocol supplementary concept word, rare disease supplementary concept word, unique identifier, synonyms, population supplementary concept word, anatomy supplementary concept word]

38. empath*.mp. [mp=title, book title, abstract, original title, name of substance word, subject heading word, floating sub-heading word, keyword heading word, organism supplementary concept word, protocol supplementary concept word, rare disease supplementary concept word, unique identifier, synonyms, population supplementary concept word, anatomy supplementary concept word]

39. interpersonal skill*.mp. [mp=title, book title, abstract, original title, name of substance word, subject heading word, floating sub-heading word, keyword heading word, organism supplementary concept word, protocol supplementary concept word, rare disease supplementary concept word, unique identifier, synonyms, population supplementary concept word, anatomy supplementary concept word]

40. intuiti*.mp. [mp=title, book title, abstract, original title, name of substance word, subject heading word, floating sub-heading word, keyword heading word, organism supplementary concept word, protocol supplementary concept word, rare disease supplementary concept word, unique identifier, synonyms, population supplementary concept word, anatomy supplementary concept word]

41. playful*.mp. [mp=title, book title, abstract, original title, name of substance word, subject heading word, floating sub-heading word, keyword heading word, organism supplementary concept word, protocol supplementary concept word, rare disease supplementary concept word, unique identifier, synonyms, population supplementary concept word, anatomy supplementary concept word]

42. Adaptation, Psychological/

43. courage/ or hope/

44. Resilience, Psychological/

45. brave*.mp. [mp=title, book title, abstract, original title, name of substance word, subject heading word, floating sub-heading word, keyword heading word, organism supplementary concept word, protocol supplementary concept word, rare disease supplementary concept word, unique identifier, synonyms, population supplementary concept word, anatomy supplementary concept word]

46. courage*.mp. [mp=title, book title, abstract, original title, name of substance word, subject heading word, floating sub-heading word, keyword heading word, organism supplementary concept word, protocol supplementary concept word, rare disease supplementary concept word, unique identifier, synonyms, population supplementary concept word, anatomy supplementary concept word]

47. entrepreneur*.mp. [mp=title, book title, abstract, original title, name of substance word, subject heading word, floating sub-heading word, keyword heading word, organism supplementary concept word, protocol supplementary concept word, rare disease supplementary concept word, unique identifier, synonyms, population supplementary concept word, anatomy supplementary concept word]

48. flexi*.mp. [mp=title, book title, abstract, original title, name of substance word, subject heading word, floating sub-heading word, keyword heading word, organism supplementary concept word, protocol supplementary concept word, rare disease supplementary concept word, unique identifier, synonyms, population supplementary concept word, anatomy supplementary concept word]

49. productiv*.mp. [mp=title, book title, abstract, original title, name of substance word, subject heading word, floating sub-heading word, keyword heading word, organism supplementary concept word, protocol supplementary concept word, rare disease supplementary concept word, unique identifier, synonyms, population supplementary concept word, anatomy supplementary concept word]

50. resilien*.mp. [mp=title, book title, abstract, original title, name of substance word, subject heading word, floating sub-heading word, keyword heading word, organism supplementary concept word, protocol supplementary concept word, rare disease supplementary concept word, unique identifier, synonyms, population supplementary concept word, anatomy supplementary concept word]

51. neurodivers*.mp. [mp=title, book title, abstract, original title, name of substance word, subject heading word, floating sub-heading word, keyword heading word, organism supplementary concept word, protocol supplementary concept word, rare disease supplementary concept word, unique identifier, synonyms, population supplementary concept word, anatomy supplementary concept word]

52. neurodiverg*.mp. [mp=title, book title, abstract, original title, name of substance word, subject heading word, floating sub-heading word, keyword heading word, organism supplementary concept word, protocol supplementary concept word, rare disease supplementary concept word, unique identifier, synonyms, population supplementary concept word, anatomy supplementary concept word]

53. 10 or 11 or 12 or 13 or 14 or 15 or 16 or 17 or 18 or 19 or 20 or 21 or 22 or 23 or 24 or 25 or 26 or 27 or 28 or 29 or 30 or 31 or 32 or 33 or 34 or 35 or 36 or 37 or 38 or 39 or 40 or 41 or 42 or 43 or 44 or 45 or 46 or 47 or 48 or 49 or 50 or 51 or 52

54. 9 and 53

PsycINFO

Initial search string (EBSCO)

(DE "attention deficit disorder with hyperactivity" OR

"attention deficit disorder with hyperactivity" OR

"attention deficit disorder" OR

"ADHD")

AND

"adult*"

AND

(DE "achievement" OR

DE "positive psychology" OR

DE "achievement motivation" OR

"strength*" OR

"positives" OR

"positive trait*" OR

"positive aspect*" OR

"positive attribute*" OR

"achievement*" OR

"positive psychology" OR

DE "enthusiasm" OR

DE "motivation" OR

DE "passion" OR

"motivat*" OR

"passion*" OR

"vig#ur*" OR

enthus* OR

DE "creativity" OR

DE "divergent thinking" OR

DE "imagination" OR

DE "problem solving" OR

"creativ*" OR

"problem solving" OR

DE "flow (consciousness state)" OR

"hyperfocus" OR

DE "curiosity" OR

DE "exploratory behavior" OR

DE "multitasking" OR

DE "imagination" OR

"curios*" OR

"multitask*" OR

DE "altruism" OR

DE "cooperation" OR

DE "communication skills" OR

DE "empathy" OR

DE "humor" OR

DE "intuition" OR

DE "social justice" OR

DE "social skills" OR

"advocat*" OR

"communi*" OR

"compassion*" OR

"interpersonal skill*" OR

"intuiti*" OR

"playful*" OR

"sense of humo*" OR

DE "adaptability (personality)" OR

DE" cognitive flexibility" OR

DE" courage" OR

DE "entrepreneurship" OR

DE "resilience (psychological)" OR

"brave*" OR

"courage*" OR

"entrepreneur*" OR

"flexi*" OR

"good in a crisis" OR

"good under pressure" OR

"productiv*" OR

"resilien*" OR

DE "neurodiversity" OR

"neurodivers*" OR

"neurodiverg*")

Updated search string (Ovid)

1.	attention deficit disorder with hyperactivity.mp. or exp Attention Deficit Disorder with Hyperactivity/

2.	attention deficit disorder.mp. or exp Attention Deficit Disorder/

3.	ADHD.mp. or exp Attention Deficit Disorder with Hyperactivity/

4.	1 and 2 and 3

5.	adult?.mp. [mp=title, abstract, heading word, table of contents, key concepts, original title, tests & measures, mesh word]

6.	positive psychology.mp. or exp Positive Psychology/

7.	achievement motivation.mp. or exp Achievement Motivation/

8.	strength?.mp. [mp=title, abstract, heading word, table of contents, key concepts, original title, tests & measures, mesh word]

9.	positives.mp. [mp=title, abstract, heading word, table of contents, key concepts, original title, tests & measures, mesh word]

10.	positive trait?.mp. [mp=title, abstract, heading word, table of contents, key concepts, original title, tests & measures, mesh word]

11.	positive aspect?.mp. [mp=title, abstract, heading word, table of contents, key concepts, original title, tests & measures, mesh word]

12.	positive attribute?.mp. [mp=title, abstract, heading word, table of contents, key concepts, original title, tests & measures, mesh word]

13.	exp Achievement/ or achievement.mp.

14.	enthusiasm.mp. or exp Enthusiasm/

15.	motivation.mp. or exp Motivation/

16.	passion.mp. or exp Passion/

17.	motivat?.mp. [mp=title, abstract, heading word, table of contents, key concepts, original title, tests & measures, mesh word]

18.	vigo?r?.mp. [mp=title, abstract, heading word, table of contents, key concepts, original title, tests & measures, mesh word]

19.	enthus?.mp. [mp=title, abstract, heading word, table of contents, key concepts, original title, tests & measures, mesh word]

20.	exp Creativity/ or creativity.mp.

21.	divergent thinking.mp. or exp Divergent Thinking/

22.	imagination.mp. or exp Imagination/

23.	problem solving.mp. or exp Problem Solving/

24.	creativ?.mp. [mp=title, abstract, heading word, table of contents, key concepts, original title, tests & measures, mesh word]

25.	"flow (consciousness state)".mp. or exp "Flow (Consciousness State)"/

26.	hyperfocus.mp. [mp=title, abstract, heading word, table of contents, key concepts, original title, tests & measures, mesh word]

27.	curiosity.mp. or exp Curiosity/

28.	exploratory behavior.mp. or exp Exploratory Behavior/

29.	exp Multitasking/ or multitasking.mp.

30.	curios?.mp. [mp=title, abstract, heading word, table of contents, key concepts, original title, tests & measures, mesh word]

31.	multitask?.mp. [mp=title, abstract, heading word, table of contents, key concepts, original title, tests & measures, mesh word]

32.	altruism.mp. or exp Altruism/

33.	exp Cooperation/ or cooperation.mp.

34.	communication skills.mp. or exp Communication Skills/

35.	empathy.mp. or exp Empathy/

36.	exp Humor/ or humor.mp.

37.	intuition.mp. or exp Intuition/

38.	social justice.mp. or exp Social Justice/

39.	social skills.mp. or exp Social Skills/

40.	advocat?.mp. [mp=title, abstract, heading word, table of contents, key concepts, original title, tests & measures, mesh word]

41.	communi?.mp. [mp=title, abstract, heading word, table of contents, key concepts, original title, tests & measures, mesh word]

42.	interpersonal skill?.mp. [mp=title, abstract, heading word, table of contents, key concepts, original title, tests & measures, mesh word]

43.	intuiti?.mp. [mp=title, abstract, heading word, table of contents, key concepts, original title, tests & measures, mesh word]

44.	playful.mp. [mp=title, abstract, heading word, table of contents, key concepts, original title, tests & measures, mesh word]

45.	sense of humo?.mp. [mp=title, abstract, heading word, table of contents, key concepts, original title, tests & measures, mesh word]

46.	"adaptability (personality)".mp. or exp "Adaptability (Personality)"/

47.	cognitive flexibility.mp. or exp Cognitive Flexibility/

48.	courage.mp. or exp Courage/

49.	entrepreneurship.mp. or exp Entrepreneurship/

50.	"resilience (psychological)".mp. or exp "Resilience (Psychological)"/

51.	brave?.mp. [mp=title, abstract, heading word, table of contents, key concepts, original title, tests & measures, mesh word]

52.	courage?.mp. [mp=title, abstract, heading word, table of contents, key concepts, original title, tests & measures, mesh word]

53.	entrepreneur?.mp. [mp=title, abstract, heading word, table of contents, key concepts, original title, tests & measures, mesh word]

54.	flexi?.mp. [mp=title, abstract, heading word, table of contents, key concepts, original title, tests & measures, mesh word]

55.	good in a crisis.mp. [mp=title, abstract, heading word, table of contents, key concepts, original title, tests & measures, mesh word]

56.	good under pressure.mp. [mp=title, abstract, heading word, table of contents, key concepts, original title, tests & measures, mesh word]

57.	productiv?.mp. [mp=title, abstract, heading word, table of contents, key concepts, original title, tests & measures, mesh word]

58.	resilien?.mp. [mp=title, abstract, heading word, table of contents, key concepts, original title, tests & measures, mesh word]

59.	exp Neurodiversity/ or neurodiversity.mp.

60.	neurodivers?.mp. [mp=title, abstract, heading word, table of contents, key concepts, original title, tests & measures, mesh word]

61.	neurodiverg?.mp. [mp=title, abstract, heading word, table of contents, key concepts, original title, tests & measures, mesh word]

62.	6 or 7 or 8 or 9 or 10 or 11 or 12 or 13 or 14 or 15 or 16 or 17 or 18 or 19 or 20 or 21 or 22 or 23 or 24 or 25 or 26 or 27 or 28 or 29 or 30 or 31 or 32 or 33 or 34 or 35 or 36 or 37 or 38 or 39 or 40 or 41 or 42 or 43 or 44 or 45 or 46 or 47 or 48 or 49 or 50 or 51 or 52 or 53 or 54 or 55 or 56 or 57 or 58 or 59 or 60 or 61

63.	4 and 5 and 62

Scopus

"attention deficit disorder with hyperactivity" OR

"attention deficit disorder with hyperactivity" OR

"attention deficit disorder" OR

"ADHD"

AND

"adult*"

AND

"strength*" OR

"positives" OR

"positive trait*" OR

"positive aspect*" OR

"positive attribute*" OR

"achievement*" OR

"positive psychology" OR

"motivat*" OR

"passion*" OR

"vig?ur*" OR

enthus* OR

"creativ*" OR

"problem solving" OR

"hyperfocus" OR

"curios*" OR

"multitask*" OR

"altruis*" OR

"cooperation" OR

"empath*" OR

"humo?r" OR

"social justice" OR

"social skill*" OR

"advocat*" OR

"communi*" OR

"compassion*" OR

"interpersonal skill*" OR

"intuiti*" OR

"playful*" OR

"adaptab*" OR

"entrepreneur*" OR

"brave*" OR

"courage*" OR

"entrepreneur*" OR

"flexi*" OR

"good in a crisis" OR

"good under pressure" OR

"productiv*" OR

"resilien*" OR

"neurodivers*" OR

"neurodiverg*"

Proquest Dissertations & Theses Global

(summary("attention deficit disorder with hyperactivity" OR "attention deficit disorder" OR "ADHD") AND "adult*" AND ("strength*" OR "positives" OR "positive trait*" OR "positive aspect*" OR "positive attribute*" OR "achievement*" OR "positive psychology" OR "motivation (psychology)" OR "motivat*" OR "passion*" OR "vigo?r*" OR "enthus*" OR "creativity" OR "creativ*" OR "problem solving" OR "hyperfocus" OR "curios*" OR "multitask*" OR "altruis*" OR "cooperation" OR "empath*" OR "humo?r" OR "social justice" OR "social skill*" OR "advoca*" OR "communi*" OR "compassion*" OR "interpersonal skill*" OR "interpersonal communication" OR "intuiti*" OR "playful*" OR "adaptab*" OR "entrepreneur*" OR "brave*" OR "courage*" OR "flexi*" OR "good in a crisis" OR "good under pressure" OR "productiv*" OR "resilien*" OR "neurodivers*" OR "neurodiverg*")) NOT (subt.exact("educational psychology" OR "developmental psychology" OR "special education" OR "education" OR "school counseling" OR "adolescents" OR "children & youth" OR "teenagers" OR "educational tests & measurements" OR "early childhood education" OR "secondary education" OR "teacher education" OR "educational leadership" OR "educational technology" OR "educational administration" OR "reading instruction" OR "child development" OR "child psychology") NOT diskw.exact("Children" OR "Adolescent" OR "Boys" OR "Child" OR "Conduct disorder" OR "Intervention" OR "Youth" OR "Childhood" OR "Girls"))

Adjustments to search

Some adjustments were made to the search strategy due to errors.

Embase

1 typo (advocat* instead of advoca*)

[https://journals.plos.org/plosone/article?id=10.1371/journal.pone.0292721#sec011](https://journals.plos.org/plosone/article?id=10.1371/journal.pone.0292721#sec011) (added to Covidence 2024-06-26)

Medline

1 typo (advocat* instead of advoca*)

*No additional resources*

PsycINFO

2 typos (advocat* instead of advoca*, vig#ur instead of vigo#r)

*No additional resources*

Scopus

2 typos (advocat* instead of advoca*, vig?ur instead of vigo?r)

*No additional resources*

Hand searching

- ADHD Guideline Development Group. (2022a). *Australian evidence-based clinical practice guideline for Attention Deficit Hyperactivity Disorder*. Australian ADHD Professionals Association.
- ADHD Guideline Development Group. (2022b). *Technical Report—Australian evidence-based clinical practice guideline for attention deficit hyperactivity disorder*. Australian ADHD Professionals Association.
- Ashinoff, B. K., & Abu-Akel, A. (2021). Hyperfocus: The forgotten frontier of attention. *Psychological Research*, *85*(1), 1–19. <http://dx.doi.org/10.1007/s00426-019-01245-8>
- Bjerrum, M. B., Pedersen, P. U., & Larsen, P. (2017). Living with symptoms of attention deficit hyperactivity disorder in adulthood: A systematic review of qualitative evidence. *JBI Database of Systematic Reviews and Implementation Reports*, *15*(4), 1080–1153. <https://doi.org/10.11124/JBISRIR-2017-003357>
  - Brock
- Champ, R. E., Adamou, M., & Tolchard, B. (2023). Seeking connection, autonomy, and emotional feedback: A self-determination theory of self-regulation in attention-deficit hyperactivity disorder. *Psychological Review*, *130*(3), 569–603. <https://doi.org/10.1037/rev0000398>
  - Dimic, Toner, Cardello
- de Schipper, E., Lundequist, A., Wilteus, A. L., Coghill, D., de Vries, P. J., Granlund, M., Holtmann, M., Jonsson, U., Karande, S., Levy, F., Al-Modayfer, O., Rohde, L., Tannock, R., Tonge, B., & Bölte, S. (2015). A comprehensive scoping review of ability and disability in ADHD using the International Classification of Functioning, Disability and Health-Children and Youth Version (ICF-CY). *European Child & Adolescent Psychiatry*, *24*(8), 859–872. <https://doi.org/10.1007/s00787-015-0727-z>
  - done (title screening of references and electronic supplementary material), no results
- Doyle, N. (2020). Neurodiversity at work: A biopsychosocial model and the impact on working adults. *British Medical Bulletin*, *135*(1), 108–125. <https://doi.org/10.1093/bmb/ldaa021>
- Dunn, S. J. (2018). *Pathology or Neurodiversity?: Cognitive Accessibility and the Rhetorical Construction of ADHD in Higher Education* [Ph.D., Purdue University]. <https://www.proquest.com/docview/2111872353/abstract/DFDEA2CE79724401PQ/1>
- Hoogman, M., Stolte, M., Baas, M., & Kroesbergen, E. (2020). Creativity and ADHD: A review of behavioral studies, the effects of psychostimulants and neural underpinnings. *Neuroscience and Biobehavioral Reviews*, *119*, 66–85.
  - Sam et al
- Levy, M. D. (2020). *The Lived Experiences of Young People with ADHD: A Review of Qualitative Literature and an Exploration of the Medicated Self* [Ph.D., New York University]. <https://www.proquest.com/docview/2454395587/abstract/A835229E39024CD4PQ/1>
- Long, G. (2022). *Understanding the Collegiate Experience for Students With ADHD* [Ed.D., South College]. <https://www.proquest.com/docview/2832728219/abstract/406E52B0D1B14853PQ/1>
  - Grotewiel
- Morellini, L., Ceroni, M., Rossi, S., Zerboni, G., Rege-Colet, L., Biglia, E., Morese, R., & Sacco, L. (2022). Social Cognition in Adult ADHD: A Systematic Review. *Frontiers in Psychology*, *13*. <https://doi.org/10.3389/fpsyg.2022.940445>
- Paek, S. H., Abdulla, A. M., & Cramond, B. (2016). A Meta-Analysis of the Relationship Between Three Common Psychopathologies—ADHD, Anxiety, and Depression—And Indicators of Little-c Creativity. *Gifted Child Quarterly*, *60*(2), 117–133. <https://doi.org/10.1177/0016986216630600>
  - Andreou & Trott
- Sarkis, E. (2014). Addressing attention-deficit/hyperactivity disorder in the workplace. *Postgraduate Medicine*, *126*(5), 25–30. <https://doi.org/10.3810/pgm.2014.09.2797>
- Shelley-Tremblay, J. F., & Rosén, L. A. (1996). Attention Deficit Hyperactivity Disorder: An Evolutionary Perspective. *The Journal of Genetic Psychology*, *157*(4), 443–453. <https://doi.org/10.1080/00221325.1996.9914877>

Grey literature

PsyRXV

500 results for ADHD, 5 saved, 3 duplicates removed

Websites

- [https://www.additudemag.com](https://www.additudemag.com/tag/survey/)
  - <https://www.additudemag.com/tag/survey/>
  - <https://www.additudemag.com/tag/adhd-research/>
    - <https://www.additudemag.com/benefits-of-adhd-crisis/>
    - <https://www.additudemag.com/no-motivation-adhd-brains-survey/>
    - <https://www.additudemag.com/what-is-your-greatest-strength-adhd-superpowers-toxic-positivity/>
    - <https://www.additudemag.com/hyperfocus-symptoms-positives-negatives-strategies/>
    - <https://www.additudemag.com/hyperfixation-adhd-stories/>
    - <https://www.additudemag.com/adhd-characteristics-benefits-strengths/>
    - <https://www.additudemag.com/slideshows/positives-of-adhd/>
- [https://adhdfoundation.org.au](https://adhdfoundation.org.au/)
  - Wiklund, J., Yu, W., Tucker, R. & Marino, L.D. (2017). ADHD, impulsivity and entrepreneurship. *Journal of Business Venturing, (32)*6, 627-656.

No results

[https://aadpa.com.au](https://aadpa.com.au/)

[https://add.org](https://add.org/)

[https://adhdeurope.eu](https://adhdeurope.eu/)

[https://chadd.org](https://chadd.org/)

<https://www.adhdsupportaustralia.com.au/>

<https://www.adhdaustralia.org.au/>
