# Supplementary material for: Attention Deficit/Hyperactivity Disorder (ADHD)-Related Strengths in Adults: A Scoping Review
Source: J Atten Disord. 2026 Mar 26;30(10):1207–32. doi: 10.1177/10870547261425737 (PMC13538910; doi:10.1177/10870547261425737)
Supplement: Supplementary file 2 [file sj-docx-2-jad-10.1177_10870547261425737.docx]

Supplementary File 2: Screening and Extraction Guidelines

| ***Inclusion*** | | ***Exclusion*** |
| --- | --- | --- |
| **Participants** | |  |
| - participants had an ADHD diagnosis, or ADHD traits (including subclinical) identified by the study   OR   - subjects with ADHD discussed by participants without ADHD (e.g., where clinicians or other stakeholders described strengths of adults with ADHD)   AND   - some/all participants/subjects aged 18 and over - co-occurring conditions can be present | | - all participants/subjects under 18 - participants/subjects did not have ADHD or ADHD traits identified by the study |
| **Concept** | |  |
| - research into strengths that are potentially related to ADHD - challenges can be discussed as long as strengths are mentioned - studies included based on topic/hypothesis, not outcomes (e.g., a study hypothesising a connection between ADHD and creativity would still be included even if no link was found) | | - no mention of strengths that may relate to ADHD (e.g., studies of mediating or moderating factors) |
| **Context** | |  |
| - any other contextual factors (e.g., gender, geographical location) - any language - no date restrictions | | - none |
| **Study characteristics** | |  |
| - experimental and quasi-experimental study designs, including randomised and non-randomised controlled trials, before-and-after studies, and interrupted time-series studies - analytical observational studies including prospective and retrospective cohort studies, case–control studies and analytical cross-sectional studies - descriptive observational study designs including case series, individual case reports and descriptive cross-sectional studies - qualitative studies, including, but not limited to, designs such as phenomenology, grounded theory, ethnography, qualitative description, action research and feminist research | | - reviews - editorials - opinion articles - other non-empirical works |
| Author | Eg, Smith; Smith & Hunt; Smith et al. (for more than 2 authors) | |
| Title | What is the title of this article, guideline, etc? Write the full title (eg, The experience of mothers and fathers in cases of stillbirth in Spain: a qualitative study) | |
| Publication | Where was this article published (eg, Midwifery; Birth; Women and Birth). If it is an organisation guideline, write the organisation (eg, American College of Obstetrics and Gynaecology). Where there may be multiple dates on an article (eg, preprints or an article made available online before it then gets published), use the date on the article that you have. | |
| Year | The year the article was published. | |
| Other publication details | Eg, volume, issue, page numbers, report number | |
| Date data were collected | The article may have collected data at another time point prior to publication. In this section write the time period (eg, 1990–2000) data were collected. If this date was not stated or no data were collected (eg, discussion paper), then write NA. | |
| Country | What country were the participants located in? | |
| Aim | What was the aim of the research, as stated by the authors? If they included research questions, include those as well. | |
| Intentionality* | Did the research have an a-priori aim to elicit ADHD-related strengths? (e.g., were strengths discussed in the introduction or was there a survey/interview question related to strengths?) | |
| Topic* | What was the broad topic of the research? | |
| Type of evidence source (primary research/conference abstract) | • Primary research: peer-reviewed research articles • Epidemiology: articles that have used population-level datasets • Conference abstracts: abstracts presented within conferences • Theses | |
| Study design | • Qualitative/Quantitative • RCT • survey • structured interview • semi-structured interview• focus group | |
| ‍Comparison group^†^ | For quantitative research, whether a comparison group was used | |
| ‍Identification of ADHD^†^ | Inclusion criteria regarding ADHD status (verified diagnosis, self-reported diagnosis, screened, and/or self-identified) | |
| Population | Inclusion criteria (eg, teachers, people not taking ADHD medication) | |
| Sample size | How many participants | |
| Age | Mean (SD) or range | |
| Gender | No. male/female/other | |
| Other demographics | Any other inclusion criteria | |
| Outcome measures/analysis | Outcome measures (quantitative research) • Thematic analysis methodology (qualitative research) | |
| Relevant results | Strength(s) identified/examined (no outcomes, statistical significance, effect sizes etc) | |

* Added as part of iterative data extraction.

† Added at the request of peer reviewers.
